# Rotaxane Formation With Intramolecular Charge Transfer Properties for Phosphate Sensing

**DOI:** 10.1002/asia.202500910

**Published:** 2025-12-07

**Authors:** Chi‐Hin Wong, Daniel Nnaemaka Tritton, Chak‐Shing Kwan, Wai‐Lun Chan, Ken Cham‐Fai Leung

**Affiliations:** ^1^ Department of Chemistry Hong Kong Baptist University Hong Kong SAR the People's Republic of China; ^2^ Department of Chemistry School of Science Great Bay University and Great Bay Institute for Advanced Study Dongguan the People's Republic of China; ^3^ The International Joint Institute of Tianjin University‐National University of Singapore in Fuzhou Tianjin University Tianjin the People's Republic of China

**Keywords:** [1]rotaxane, anion sensing, fluorescence quenching, intramolecular charge transfer, phosphate

## Abstract

Mechanically interlocked molecules (MIMs), specifically rotaxanes, have been demonstrated to have immense utility as sensing materials with a wide array of analytes, owing to their unique topologies and properties afforded by the presence of a mechanical bond. Among other more conventional sensor compounds, aromatic amide functionalities have been employed to induce intramolecular charge transfer (ICT) processes, where a crown ether donor oxygen atom donates electron density to an aromatic amide acceptor, resulting in reduced or no emission; this methodology has received less attention for its application in rotaxanes, particularly to detect anionic species. Hence, in this work, we were inspired to construct a novel kinetically stable [1]rotaxane comprising dibenzo[24]crown‐8 donor and *N*‐benzylbenzamide acceptor moieties, namely 1‐H(Rot)·PF_6_, using a template‐directed “slippage” approach. Before [1]rotaxane formation, the linear molecule showed ICT response. Fluorescence response was restored after the [1]rotaxane formed as the dialkylammonium group threaded through the crown ether, inhibiting ICT from the oxygen atoms to the aromatic amide. The [1]rotaxane showed marginal fluorescence quenching when titrated with different metal cations by a photoinduced electron transfer (PeT) mechanism; however, when titrated against various inorganic anions, 1‐H(Rot)·PF_6_ exhibited substantial emission quenching (up to ca. 50%) in the presence of phosphate (PO_4_^3‒^).

## Graphical abstract

“ON‐OFF” fluorescence array in [1]rotaxane formation for selective phosphate chemosensing.

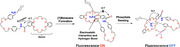

## 1 Introduction

[*n*]Rotaxanes, where *n* is the number of subcomponents, are the quintessential example of mechanically interlocked molecules (MIMs) which feature a dumbbell‐shaped linear compound, termed the “thread”/“axle” woven through a cyclic moiety referred to as the “macrocycle” [1, 2, 3], the threads are fitted with bulky functional groups called “stoppers” which help to prevent the macrocycle, which typically comprises a polyether crown with a specific number of heteroatoms including nitrogen (N) and oxygen (O), from “slipping” off the thread, which would otherwise dissociate the structure and terminate the mechanical bond holding the supramolecular architectures together [4, 5, 6]. It should be noted that whilst polyethers are the most widely used macrocycle building blocks in rotaxanes, many other types are commonly employed, both natural and synthetic, including cyclodextrins (CDs) [7], cucurbiturils (CBs) [8], calixarenes [9, 10], pillar[n]arenes [11], and tetralactams (Leigh‐type or Hunter/Vögtle‐types) [12, 13]. Owing to their diverse and robust architectures, rotaxanes have found a common place in the development of molecular machines [14, 15, 16], sensors [17, 18, 19], “smart” materials [20, 21, 22], and novel chiral compounds [23, 24, 25]. Among the various types available for synthesis in the supramolecular chemist's toolkit, [1]rotaxanes have garnered particular interest in recent years as they consist of a single molecule threaded through itself, which leads to unique properties and topologies [26, 27, 28, 29]. In terms of the synthetic methodology, a wide array of strategies have been used to prepare such [1]rotaxanes, including clipping [30], capping [31], active metal template synthesis [32], and self‐entangling [28, 33].

Delving into one specific example of their various applications, rotaxanes have been exploited as fluorescent chemosensors for “off/on” detection of numerous analyte ions, including cations [34, 35, 36, 37] and anions [38, 39, 40, 41]. Focusing on negatively charged species, anions play vital roles in biological [42, 43, 44, 45] and environmental processes [46, 47, 48], which has attested to the rapid growth of the field of anion recognition in recent years. The development of intricate sensors for rapid and sensitive detection of selected anions is gaining attraction, and rotaxanes are well‐suited to this desired role thanks to their pre‐organized cavities for analyte binding [49].

**SCHEME 1:**
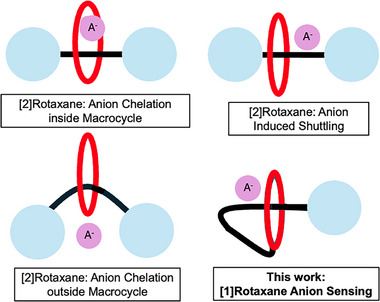
Mechanically interlocked synthetic strategies for anion (A‒) detection: Reported [2]rotaxane‐based (a) anion chelation on macrocycle; (b) anion‐induced shuttling; (c) anion chelation outside macrocycle; and (d) This work: [1]Rotaxane anion sensing.

**FIGURE 1:**
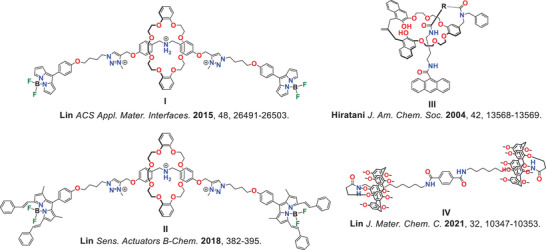
Chemical structures of reported rotaxanes for anion/cation sensing.

MIMs’ syntheses usually require a template, including anions (Scheme 1a) and non‐covalent interactions such as H‐bonding and π‐donor/acceptor [50, 51]. Focusing on anions, the removal of the anion template yields an interlocked host. This host contains a unique, rigid three‐dimensional cavity that demonstrates high selectivity for complementary anionic guests, which can selectively sense anionic guests [37, 42, 52]. Depending on the number of recognition sites/stations, the components in certain MIMs can reversibly change shape, which is governed by the system's thermodynamics alone or induced by an external stimulus (Scheme 1b,c). This dynamic behavior makes MIMs excellent candidates for building molecular machines and switches. Researchers have constructed several MIMs that change shape in the presence of anions, making them effective optical sensors; these sensors generally contain a “reporter” group that produces a measurable fluorescent response to a specific target molecule/analyte, by changes in the sensor's molecular structure [53]. Typically, these systems are multi‐station rotaxanes. They function by having a ring component shuttling away from a photoactive site to a station that binds the anion, a movement that produces a clear photophysical response. Among those, Lin and co‐workers prepared a dihydrogen phosphate (H_2_PO_4_^‒^) controllable [2]rotaxane with a bis triazolium thread stoppered by two boron‐dipyrromethene (BODIPY) fluorophores (I in Figure 1) [54]. This BODIPY‐terminated [2]rotaxane exhibited a strong green fluorescence, which, upon the addition of H_2_PO_4_^‒^ led to fluorescence quenching due to proposed interactions of the anion with the triazolium groups; ^1^H NMR titrations and quantum mechanical calculations suggested the triazolium moieties were potential receptors for H_2_PO_4_^‒^ thanks to the rotaxane supposedly folding to yield a cage‐like structure. Quenching of the fluorescence emission was attributed to photoinduced electron transfer (PeT) between the binding site of H_2_PO_4_^‒^ and the excited state of the BODIPY moiety. Furthermore, controllable acid‐base fluorescence turn‐on/off was achieved by macrocycle shuttling between the ammonium station (with acid) and the triazolium group (with base). The same group synthesized a far‐red emitting BODIPY‐terminated [2]rotaxane (II in Figure 1) by the same concept for selective H_2_PO_4_^‒^ sensing with strong red fluorescence [55]. Up to that point, examples of a far‐red emitting rotaxane with acid‐base and H_2_PO_4_^‒^ responsive properties had not been reported, showcasing the advantages of combining far‐red fluorescing moieties into MIMs. Significantly, both I and II showed utility in detecting H_2_PO_4_^‒^ in specific cell lines, which is of importance given that H_2_PO_4_^‒^ is the most abundant physiological anion and plays key roles in energy storage and signal transduction [56]. To the best of our knowledge, I and II are the only reported examples of rotaxane sensors for selective H_2_PO_4_^‒^ detection.

Despite the vast MIM cation and anion sensor examples, the sensing ability of [1]rotaxanes (Scheme 1d) are relatively unexplored. Hiratani and co‐workers utilized a [1]rotaxane with an anthracene reporting group (III in Figure 1) for selective lithium cation (Li^+^) sensing [57]. Upon the binding of Li^+^ within the macrocycle, it enhanced the electron transfer from naphthalene to anthracene, thus enhancing a blue fluorescence. Lin group prepared a pillar[5]arene‐based gemini‐[1]rotaxane (IV in Figure 1) for selective L‐Arginine (L‐Arg) sensing [58]. L‐Arg bonded to the amide groups on the gemini‐[1]rotaxane and induced the pillar[5]arene shuttling, displaying a Tyndall phenomenon, which revealed blue fluorescence.

From a design perspective, fluorescence reporter groups‐containing aromatic amide moieties have been utilized to control emission by inhibiting photoinduced intramolecular charge transfer (ICT) upon metal ion binding [59, 60, 61]; ICT is a photoexcitation process between an electron donor and electron acceptor moiety linked by a single bond, in which intramolecular electron transfer occurs, resulting in a typically non‐emissive charge separated state [62]. Most of the examples employing the aromatic amide synthetic strategy focus on the detection of cations by inhibiting ICT relaxation, which provides an opportunity to develop novel ICT sensing materials for important anion species such as PO_4_^3‒^ [63], with improved efficiency and selectivity.

Based on the above discussion, we were inspired to develop an original [1]rotaxane, namely **1‐H(Rot)·PF_6_**, which is kinetically stable and prepared using a template‐directed “slippage” approach (Scheme 1d) [6]. Once the [1]rotaxane was formed, it gained kinetic stability and was relatively stable at elevated temperatures throughout the characterization of sensing studies. Therefore, we classify our as‐synthesized compound as a [1]rotaxane instead of a [1]pseudorotaxane. The fluorophore in this [1]rotaxane is a *N*‐benzylbenzamide moiety; whilst the basic benzamide structure is typically considered as weakly fluorescent, its derivatives may function as efficient fluorophores by rational design to induce radiative decay [64, 65]. Initially the MIM inhibited ICT quenching from the crown ether oxygen atoms to the benzylamide, maintaining fluorescence output; negligible fluorescence quenching was observed in the presence of 20 different metal cations; however, most significantly, the supramolecule showed selective quenching for PO_4_^3‒^ over 6 other common inorganic anions, rendering **1‐H(Rot)·PF_6_** as a selective “turn‐off” sensor for PO_4_^3‒^.

## 2 Results and Discussion

**SCHEME 2:**
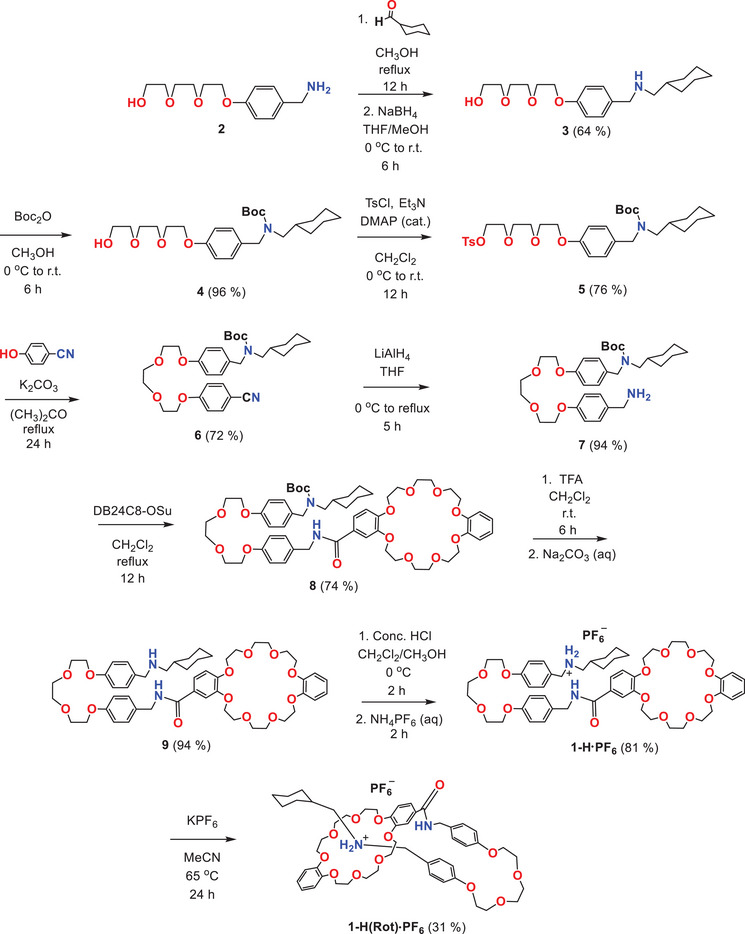
Synthetic scheme of compounds and **1‐H(Rot)·PF_6_**.

**FIGURE 2:**
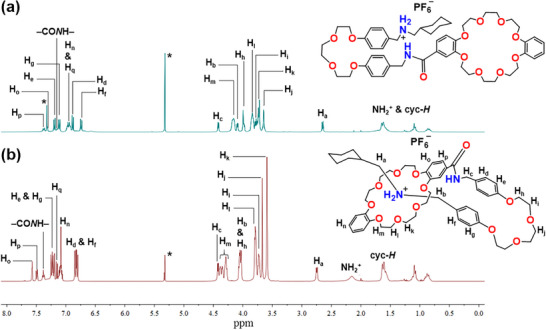
Stacked ^1^H NMR spectra (400 MHz, CD_2_Cl_2_, 298 K) of (a) **1‐H·PF_6_** and (b) **1‐H(Rot)·PF_6_**. * = solvent residual signal.

**FIGURE 3:**
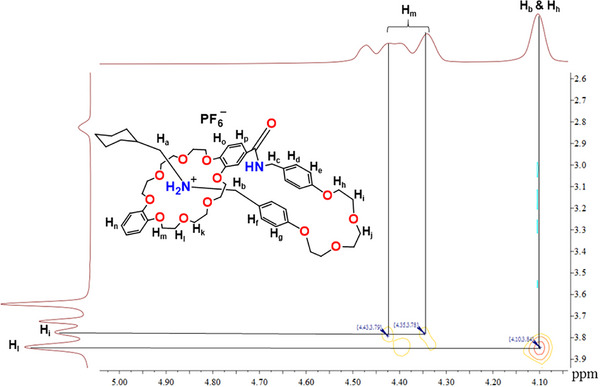
Partial 2D NOESY spectrum (400 MHz, CD_2_Cl_2_, 298 K) of **1‐H(Rot)·PF_6_**.

The [1]rotaxane **1‐H(Rot)·PF_6_** was prepared in 9 steps according to Scheme 2. Primary amine compound **2** was first stoppered with cyclohexane to afford secondary amine **3** before protecting the amine with *tert*‐butoxycarbonyl (Boc) to give **4**. Terminal OH group in **4** was tosylated with *p*‐toluenesulfonyl chloride to **5** before undergoing a Williamson ether reaction with 4‐cyanophenol to give a cyano compound **6**, then reducing the cyano group to a primary amine compound **7**. Compound **7** was coupled with dibenzo [24]crown‐8 succinimide (DB24C8‐OSu) to form **8,** which then underwent trifluoroacetic acid (TFA) Boc deprotection to **9,** followed by treatment with conc. HCl/sat. NH_4_PF_6_ solution to generate the salt **1‐H·PF_6_**. The synthesis culminated in incubating **1‐H·PF_6_** with KPF_6_ in CH_3_CN to give the final rotaxane **1‐H(Rot)·PF_6_** by a slippage method. Stacked ^1^H NMR spectra of compound **1‐H·PF_6_** and the [1]rotaxane **1‐H(Rot)·PF_6_** are presented in Figure 2. All signals have been assigned to the protons of each compound and there is a clear shift of the peaks in **1‐H(Rot)·PF_6_**, indicating the successful formation of the rotaxane: first, the aromatic protons H_e_, H_f_ and H_g_ show a downfield shift, whilst H_d_ moves up‐field, indicating a displaced “edge‐on” π‐π interaction between the corresponding phenyl groups on the rotaxane thread [66]. Additionally, the proton signals of H_a_ and H_b_, located on the methylene moieties adjacent to the ammonium (NH_2_^+^) station, shifted from 2.65/4.09 ppm to 2.75/4.01 ppm, respectively. Notably, the ethylene glycol proton H_m_ shifted downfield and changed its peak pattern, attributed to the hydrogen bond/electrostatic interaction between the crown ether and dialkyl NH_2_^+^ moieties (Figure 2). To further investigate the structure of **1‐H(Rot)·PF_6_**, a 2D Nuclear Overhauser Effect Spectroscopy (NOESY) NMR analysis was performed (Figures 3 and S20). Figure S20 shows the full NOE spectrum of the product, while Figure 3 shows the partial spectrum. Correlation peaks (Figure 3) were observed between the DB24C8 protons H_m_/H_l_ and tri‐ethylene glycol protons H_i_/H_h_ and ‒NH_2_^+^‒C*H*_2_ *‒* proton H_b_, attesting that the glycol chain was folded and threaded through the crown ether macrocycle, further confirming the [1]rotaxane formation.

Figure S20 also revealed that the NCH_2_ (H_b_) protons correlate to aromatic protons H_d_ (H_b_/H_d_) and H_e_ (H_b_/H_e_). Proton H_a_ also showed correlations with NH_2_ and H_n_ protons.

**FIGURE 4:**
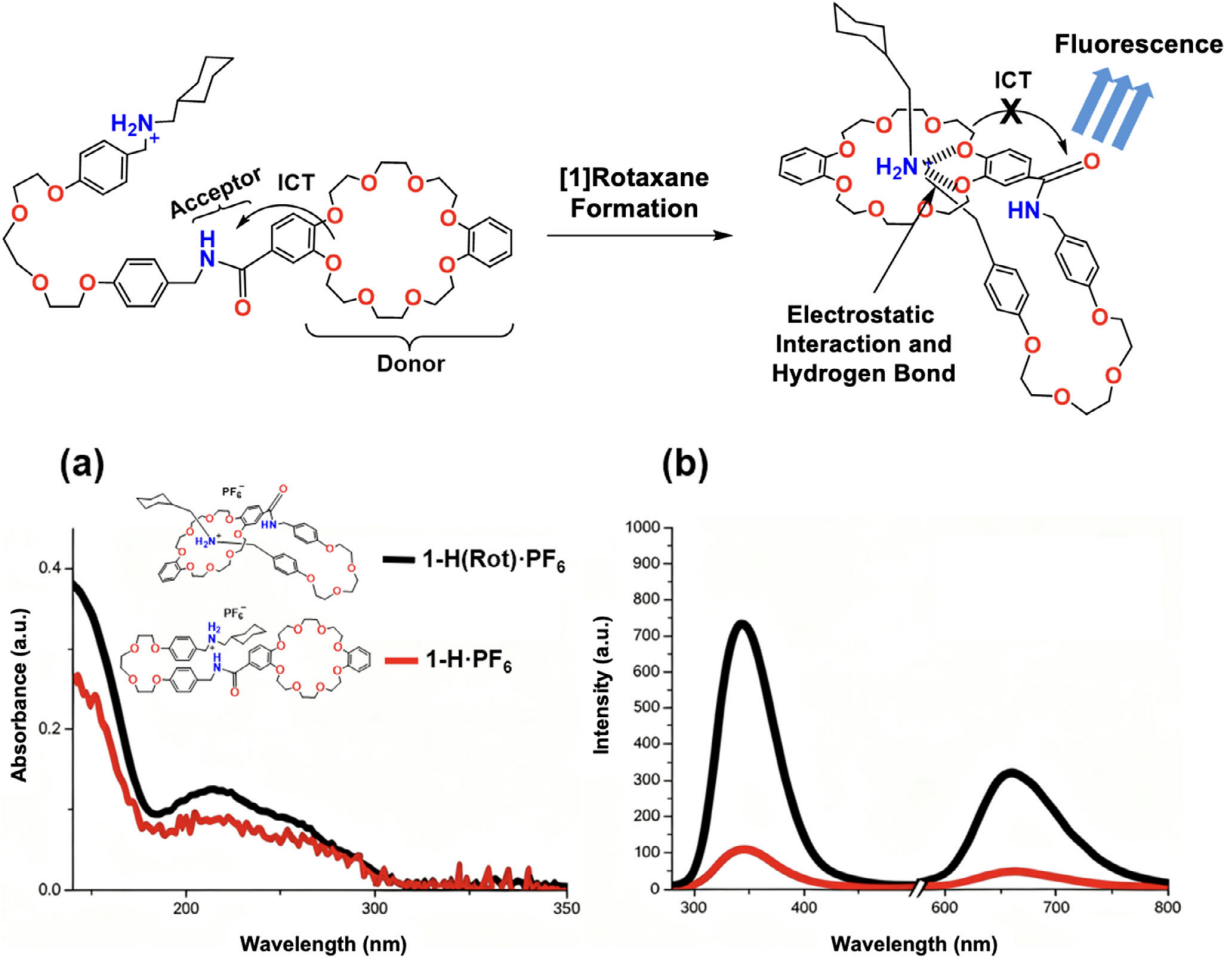
(above) Fluorescence enhancement mechanism in **1‐H(Rot)·PF_6_**. (below) (a) UV–vis absorbance profile of **1‐H·PF_6_** and **1‐H(Rot)·PF_6_**. (b) Fluorescence emission profile (*λ*_ex_ 270 nm) of **1‐H·PF_6_** and **1‐H(Rot)·PF_6_**. UV and fluorescence run in CH_3_CN (0.01 mM) with slit width 10, 10 nm.

The UV absorption spectra of compound **1‐H·PF_6_** and its [1]rotaxane form **1‐H(Rot)·PF_6_** are shown in Figure 4a. Both compounds exhibit a UV absorption with similar‐shaped bands in the region of 240‒300 nm, which is characteristic of molecules containing an aromatic amide moiety in their structures [67]. On the other hand, the emission spectra at an excitation wavelength of 270 nm of the two compounds are shown in Figure 4b. Both compounds show a *λ*_max_ at 345 nm and a smaller band at 656 nm, which are ascribed to normal locally excited state (LE) F_1_ fluorescence and a unique excited ICT F_2_ species, respectively, possibly attributed to a twisted intramolecular charge transfer (TICT) process [68, 69]. The intensity of the latter F_2_ band was smaller in the relatively polar CH_3_CN solvent, a phenomenon observed in a previous related study [69]. The emission intensity of the normal F1 and atypical F2 bands for **1‐H(Rot)·PF_6_** is ca. 7.5‐fold and 7‐fold higher than that of compound **1‐H·PF_6_**, respectively, owing to finely controlled intramolecular ICT quenching. In **1‐H·PF_6_**, the crown oxygen atoms are available to donate electron density to the benzamide and hence lead to fluorescence quenching due to non‐radiative ICT (Figure 4) [62, 70]. Contrastingly, the macrocycle ether oxygen(s) in the [1]rotaxane likely experience electrostatic interaction and hydrogen bonding by the mechanical bond formation, which plays a fundamental part in controlling ICT; upon rotaxane formation, the crown ether oxygens are hydrogen bonded with the NH_2_^+^ station and electron donation to benzamide is restricted, ICT is no longer dominant and thus fluorescence is turned “ON” (Figure 4) [71]. Furthermore, the second emission band at *λ*_max_ 656 nm is ascribed to the fluorescence emission of **1‐H(Rot)·PF_6_**, with a lifetime of 4.41 ns.

**FIGURE 5:**
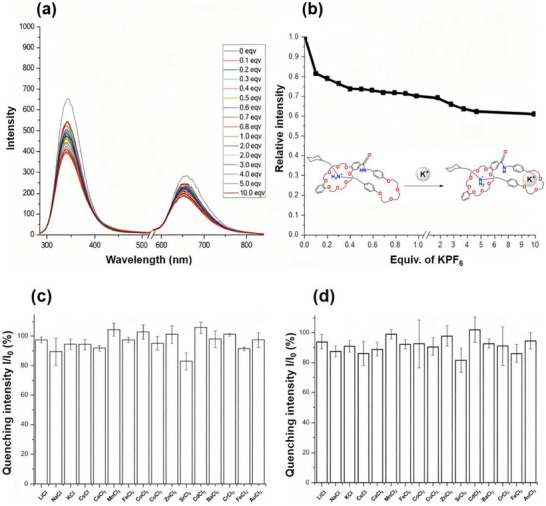
(a) Stacked fluorescence spectra of **1‐H(Rot)·PF_6_** with varying equivalents of KPF_6_; (b) plot of KPF_6_ equiv. vs. relative emission intensity (*λ*_ex_ 345 nm). All fluorescence runs in CH_3_CN (0.01 mM) with a slit width of 10, 10 nm. (c) plot of quenching intensity (%) of **1‐H(Rot)·PF_6_** with various metal ions for 30 min; *I* = intensity of metal ion treated **1‐H(Rot)·PF_6_** and *I*_0_ = H(Rot)·PF_6_ intensity. (d) plot of quenching intensity (%) of **1‐H(Rot)·PF_6_** with various metal ions for 24 h. Fluorescence run in CH_3_CN (0.01 mM) with 1 equiv. of all metal ions.

One of the possible alternative methods to induce fluorescence quenching is the introduction of a metal ion for competitive binding with the crown ether macrocycle. The fluorometric titration of **1‐H(Rot)·PF_6_** with KPF_6_ is shown in Figure 5a,b, revealing that the emission intensity did not showcase a significant quenching effect. This is attested by the fact that the crown ether was still able to template around the NH_2_^+^ station, thereby stopping ICT quenching by the mechanically interlocked section of the compound. Despite the emission intensity only falling to 70% of the initial output emission, the signal decrease was presumably due to K^+^ sited on to the smaller podant arm containing an oligoethylene glycol, which features less significant donor character than the crown ether moiety, causing an insignificant quenching on the [1]rotaxane. To further investigate the metal ion quenching effect, **1‐H(Rot)·PF_6_** was tested with 16 different metal cation species (Figure 5c,d). There was minimal emission intensity quenching with the metal ions (ca. 0‒20%) following incubation for 30 min and 24 h. It is assumed that the size/charge of the metal ions has an insignificant role in distorting the mechanical bond, as well as restoring ICT quenching. It is important to note that fluorescence quenching by metal cations in this system could also be accounted for by complexation in the [53] crown‐8 macrocycle, thereby competing with the ammonium station; further experiments are underway to investigate the potential of this [1]rotaxane as a metal ion sensor (Figure 5).

**FIGURE 6:**
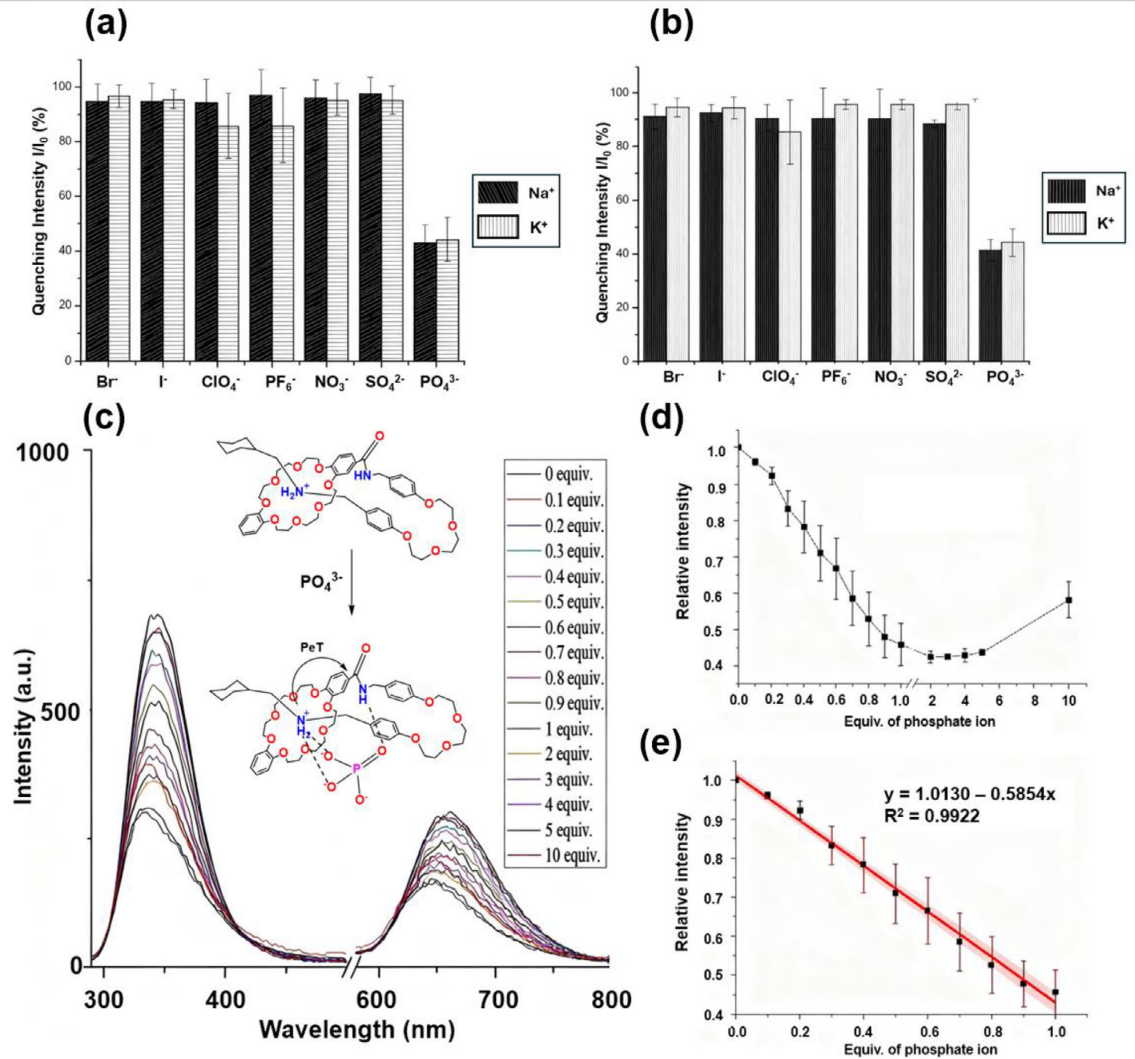
Plot of relative emission intensity quenching of **1‐H(Rot)·PF_6_** after treatment with various anions for (a) 30 min and (b) 24 h, where I = intensity of metal ion‐treated **1‐H(Rot)·PF_6_** and *I*_0_ = **H(Rot)·PF_6_** intensity. Fluorescence run in CH_3_CN (0.01 mM) with 1 equiv. of all anions; (c) stacked emission spectra of **1‐H(Rot)·PF_6_** with varying amounts of Na_3_PO_4_; (d) plot of relative emission intensity (*λ*_ex_ 345 nm) against equiv. of Na_3_PO_4_; (e) plot of relative emission intensity (*λ*_ex_ 345 nm) against the first equivalent of Na_3_PO_4_. All fluorescence runs in CH_3_CN (0.01 mM) with a slit width of 10, 10 nm.

To test the anion‐sensing capability, **1‐H(Rot)·PF_6_** was titrated against seven different inorganic anions by fluorescence spectroscopy. Only PO_4_^3‒^ elicited a substantial quenching of emission after 30 min (Figure 6a) and 24 h (Figure 6b). Using either K^+^ or Na^+^ as the counter cation did not generate a significant difference, due to the fact that PO_4_^3‒^ experiences hydrogen bonding with the NH_2_^+^ station and amide proton, therefore distorting the mechanical bonding interaction between NH_2_^+^ and the crown ether [72]. Consequently, PO_4_^3‒^ induced fluorescence emission quenching presumably because of the interaction of PO_4_^3‒^ with the ammonium and amide moieties, leading to PeT from the bounded ammonium and crown ether's oxygen atoms to the amide, which was facilitated by their proximity in the [1]rotaxane form; further to this, the system was able to reach equilibrium after 30 min, allowing for instant PO_4_^3‒^ detection. For the fluorescence titration of **1‐H(Rot)·PF_6_** with Na_3_PO_4_ (Figure 6c), the emission intensity varied with increasing amount of Na_3_PO_4_ and showed a linear relationship with a good correlation value up to 1 equiv. (Figure 6e). The emission plateaued at 2–5 equiv., after which emission increased with increasing water content (Figure 6d).

## 3 Conclusion

An ammonium template‐directed slippage strategy from **1‐H·PF_6_** was employed to successfully obtain the novel [1]rotaxane **1‐H(Rot)·PF_6_**. Fluorescence spectrometric studies (from “OFF” to “ON”) revealed that the compound emission was enhanced upon rotaxane formation, thanks to the mechanically interlocked structure behaving as a barrier to the ICT quenching pathway. When tested against 16 different metal cations with the [1]rotaxane, only minor emission quenching (ca. 0‒20%) was observed. However, when investigated with 7 different common inorganic anions, it was specifically PO_4_^3‒^ that notably generated a significant quenching effect with a linear relationship and a decent correlation value for the plot of PO_4_^3‒^ equiv. vs. relative emission intensity. The fluorescence spectrometric results (from “ON” to “OFF”) indicate **1‐H(Rot)·PF_6_** is a selective sensor for phosphate via a PeT mechanism and contributes to the “ON‐OFF” sensing array, which shows enhanced selectivity towards environmentally and biologically significant ions.

## 4 Experimental Section

The synthetic procedures and characterization data of the compounds (^1^H NMR, ^13^C NMR, HRMS) can be found in the supporting information (Figures S1‒S20). Partial experimental procedures and raw data were obtained from the repository [73].

## Conflicts of Interest

The authors declare no conflicts of interest.

## Supporting information




**Supporting file**: asia70498‐sup‐0001‐SuppMat.pdf

## Data Availability

The data that support the findings of this study are available in the supplementary material of this article.
