# Supplementary material for: Rotaxane Formation With Intramolecular Charge Transfer Properties for Phosphate Sensing
Source: Chem Asian J. 2025 Dec 7;21(1):e00910. doi: 10.1002/asia.202500910 (PMC12802827; doi:10.1002/asia.202500910)
Supplement: Supplementary file 1 — Supporting file: asia70498‐sup‐0001‐SuppMat.pdf [file ASIA-21-e00910-s001.pdf]

Supporting Information

[1]Rotaxane Formation with Intramolecular Charge Transfer Properties for Phosphate Sensing

Chi-Hin Wong,‡[a] Daniel Nnaemaka Tritton,‡[a] Chak-Shing Kwan,[a,b,]* Wai-Lun Chan,[a,c,]* and Ken Cham-Fai Leung[a,]*

[a] Department of Chemistry, Hong Kong Baptist University, Kowloon Tong, Kowloon, Hong Kong SAR, P. R. China
E-mail: cfleung@hkbu.edu.hk

[b] Department of Chemistry, School of Science, Great Bay University and Great Bay Institute for Advanced Study, Dongguan 523000, P. R. China
E-mail: cskwan@gbu.edu.cn

[c] The International Joint Institute of Tianjin University-National University of Singapore in Fuzhou, Tianjin University, Tianjin 300072, P. R. China
Email: kwlchan@nus.edu.sg

‡ These authors contributed equally to this work.

Table of Contents

1. General information S2

2. **1-H(Rot)·PF$_6$** preparation S3

3. NMR spectra of new compounds S14

4. Mass spectrum of **1-H(Rot)·PF$_6$** S25

5. References S26

1. General information

All reagents and dry solvents were bought from commercial sources and used without further purifying. Reactions were performed under nitrogen unless otherwise stated. Chromatography purifications were performed on silica gel (SiO_2) with the indicated eluents. Deionized water was obtained from Milli-Q ICW3000 water system. ^1H and ^{13}C NMR spectra for structural characterization were recorded with Bruker Avance 400 (^1H: 400 MHz; ^{13}C: 101 MHz) spectrometer at 297 K. All NMR samples were prepared in $CDCl_3$ unless otherwise stated. Spectra were calibrated internally using the CH_2Cl_2 residual peak in CD_2Cl_2 (^1H: $\delta = 5.32$; ^{13}C: $\delta = 54.0$ ppm) and the $CHCl_3$ residual peak in $CDCl_3$ (^1H: $\delta = 7.26$; ^{13}C: $\delta = 77.2$ ppm). Chemical shifts were reported as parts per million (ppm) in δ scale and coupling constants (*J*) were reported in hertz. Matrix-assisted laser desorption/ionization time of flight (MALDI-TOF) mass spectra were measured on a Bruker SolariX 9.4T mass spectrometer. The reported molecular mass (*m/z*) values correspond to the most abundant monoisotopic masses. UV-Vis spectra were obtained by Agilent UV-Vis spectrometer Cary 300. Fluorescence spectra were obtained by Perkin Elmer LS 55 fluorescence spectrometer. For titration experiments, the concentration of the [1]rotaxane was fixed at 0.01 mM in dry CH_3CN (1 mL) and adding one-time with various metal salts (1:1 stoichiometry) saturated aqueous solution for 30 mins. All fluorescence run relative emission intensity (λ_{ex} 345 nm) in CH_3CN (0.01 mM) with slit width 10, 10 nm.

2. 1-H(Rot)·PF_6 preparation[1]

Scheme 2 (main text) shows the synthesis of **1-H(Rot)·PF_6**. Amine compound **2** was prepared with high yield and purity according to literature.[2] Amine compound **3** was prepared first by imine formation between amine **2** and cyclohexanecarboxaldehyde in CH_3OH under reflux for 12 h, followed by the addition of $NaBH_4$ to reduce the imine into an amine. Compound **3** was obtained with an overall 64 % yield in two steps. The secondary amine compound **3** was protected with a Boc group with di-*tert*-butyl dicarbonate to afford **4** in 96 % yield. Boc-protected compound **4** was then tosylated with *p*-toluenesulfonyl chloride to give compound **5** in 76 % yield. Subsequently, compound **5** was reacted through Williamson ether synthesis with 4-cyanophenol to prepare compound **6** in 72 % yield. Cyano-compound **6** was then reduced by $LiAlH_4$ to obtain a primary amine **7** in 94 % yield. Afterwards, Compound **7** was coupled with dibenzo[24]crown-8 succinimide (DB24C8-OSu) to obtain compound **8** with 74 % yield. Compound **9** was prepared by deprotecting the Boc-protected compound **8** with TFA in CH_2Cl_2 with 94 % yield. Compound **1-H·PF_6** was prepared by protonating compound **9** with conc. HCl to pH 3, followed by counterion exchange with sat. NH_4PF_6 solution with 81 % yield. Finally, [1]rotaxane **1-H(Rot)·PF_6** was prepared by incubating compound **1-H·PF_6** with KPF_6 in CH_3CN with 32 % yield through a slippage approach. **1-H·PF_6** was synthesized in 9 steps with an overall 6 % yield.

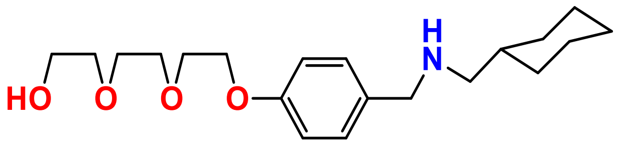

Synthesis of compound **3**

Compound **3** was prepared by a reductive amination. A solution of compound **2** (12.67 g, 49.6 mmol) and cyclohexanecarboxaldehyde (5.84 g, 52.1 mmol) in CH_3OH (50 mL) was heated at reflux for 12 h. The resultant mixture was evaporated to dryness and the residue was re-dissolved in 30 mL anhydrous THF/CH_3OH (2:1). $NaBH_4$ (2.82 g, 74.4 mmol) was then added to the solution at 0 ºC and stirred for 6 h. The resulting solution was dried in vacuum and re-dissolved in $CHCl_3$ (50 mL). The solution was then washed with H_2O (3 × 25 mL). The organic extract was dried over anhydrous $MgSO_4$, and the resulting mixture was filtered through a pack of celite. The filtrate was evaporated to dryness. Flash column chromatography with EtOAc on silica gel of the residue gave the compound **3** (11.16 g, 64 %) as a pale-yellow liquid. R_f: 0.21 (EtOAc). ^{1}H NMR (400 MHz, $CDCl_3$): δ = 7.25 – 7.18 (m, 2H, Ar*H*), 6.92 – 6.83 (m, 2H, Ar*H*), 4.15 – 4.09 (m, 2H, CH_2NH), 3.85 (dd, *J* = 5.4, 4.2 Hz, 2H, CH_2O–), 3.75 – 3.67 (m, 8H, CH_2O–), 3.63 – 3.57 (m, 2H, CH_2O–), 2.43 (d, *J* = 6.7 Hz, 2H, CH_2NH), 2.13 (s, 2H, –N*H* & –O*H*), 1.77 – 1.61 (m, 5H, cyc-*H*), 1.47 (ddd, *J* = 11.3, 6.8, 3.4 Hz, 1H, cyc-*H*), 1.29 – 1.08 (m, 3H, cyc-*H*), 0.95 – 0.83 (m, 2H, cyc-*H*). ^{13}C NMR (100 MHz, $CDCl_3$): δ = 157.8, 133.0, 129.4, 114.7, 77.5, 77.2, 76.8, 72.7, 71.0, 70.5, 69.9, 67.6, 61.9, 56.2, 53.58, 37.99, 31.60, 26.82, 26.20. HRMS (MALDI-TOF): $C_{20}H_{34}NO_4$ $[M+H]^+$: calcd 352.2482; found 352.2463.

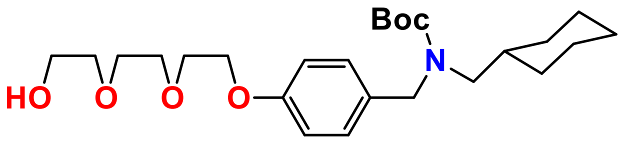

Synthesis of compound **4**

A solution of compound **3** (11.16 g, 31.8 mmol) and di-*tert*-butyl dicarbonate (7.00 g, 32.1 mmol) in CH_3OH (40 mL) was stirred at 0 ºC for 1 h and continued stirring at ambient temperature for another 5 h. The resulting solution was dried in vacuum and the residue was partitioned between dilute HCl (0.1 M, 50 mL) and EtOAc (25 mL). The aqueous layer was further extracted with EtOAc (3 × 25 mL). The combined organic layer was dried over anhydrous $MgSO_4$ and the filtrate was evaporated to dryness. Flash column chromatography with n-hex/EtOAc (1:1) on silica gel of the residue gave compound **4** (13.75 g, 96 %) as a colorless liquid. R_f: 0.61 (n-hex/EtOAc = 1:1). ^{1}H NMR (400 MHz, $CDCl_3$) δ = 7.11 (t, *J* = 10.0 Hz, 2H, Ar*H*), 6.84 (d, *J* = 8.6 Hz, 2H, Ar*H*), 4.34 (d, *J* = 21.1 Hz, 2H, C*H*$_2$NH), 4.17 – 4.06 (m, 2H, C*H*$_2$O–), 3.89 – 3.79 (m, 2H, C*H*$_2$O–), 3.77 – 3.64 (m, 6H, C*H*$_2$O–), 3.62 – 3.53 (m, 2H, C*H*$_2$O–), 2.96 (dd, *J* = 38.2, 5.9 Hz, 2H, C*H*$_2$NH), 2.69 (s, 1H, –O*H*), 1.74 – 1.54 (m, 6H, cyc-*H*), 1.44 (d, *J* = 20.3 Hz, 9H, C*H*$_3$), 1.29 – 1.06 (m, 3H, cyc-*H*), 0.88 (s, 2H, cyc-*H*). ^{13}C NMR (100 MHz, $CDCl_3$) δ = 157.8, 156.4, 156.0, 131.1, 130.9, 129.0, 128.4, 114.6, 79.5, 77.5, 77.2, 76.8, 72.6, 70.9, 70.4, 69.8, 67.4, 61.8, 52.5, 52.2, 50.5, 49.5, 36.9, 36.6, 31.0, 28.6, 26.6, 26.0. HRMS (MALDI-TOF): $C_{25}H_{41}NO_6Na$ $[M+Na]^+$: calcd 474.2826; found 474.2796.

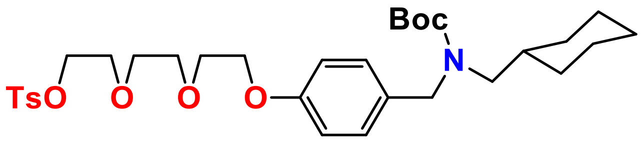

Synthesis of compound **5**

A solution of compound **4** (13.75 g, 30.4 mmol), Et_3N (12.7 mL, 91.3 mmol) and DMAP (cat.) in CH_2Cl_2 (100 mL) was stirred at 0 ºC. A solution of TsCl (6.09 g, 31.9 mmol) in CH_2Cl_2 (50 mL) was added dropwise to the above mixture at 0 ºC. The mixture was stirred for another 12 h at ambient temperature. The resulting solution was evaporated to dryness, and the residue was partitioned between dilute HCl (0.1 M, 100 mL) and CH_2Cl_2 (200 mL). The organic layer was further washed with dilute HCl (3 × 50 mL), H_2O (3 × 50 mL) and sat. NaCl solution (3 × 25 mL). The organic layer was dried over anhydrous $MgSO_4$, and the mixture was filtered with a pack of celite. The filtrate was evaporated to dryness. Flash column chromatography with n-hex/EtOAc (3:1) on silica gel of the residue gave the compound **5** (14.02 g, 76 %) as a pale-yellow liquid. R_f: 0.67 (n-hex/EtOAc = 3:1). ^{1}H NMR (400 MHz, $CDCl_3$) δ = 7.77 (dd, J = 8.3, 1.6 Hz, 2H, ArH), 7.31 (d, J = 7.6 Hz, 2H, ArH), 7.11 (s, 2H, ArH), 6.83 (d, J = 8.0 Hz, 2H, ArH), 4.34 (d, J = 20.4 Hz, 2H, CH_2NH), 4.18 – 4.11 (m, 2H, CH_2O–), 4.07 (s, 2H, CH_2O–), 3.79 (s, 2H, CH_2O–), 3.70 – 3.56 (m, 6H, CH_2O–), 2.96 (d, J = 32.8 Hz, 2H, CH_2NH), 2.41 (s, 3H, ArCH_3), 1.65 (dd, J = 22.5, 9.1 Hz, 6H, cyc-H), 1.44 (d, J = 20.1 Hz, 9H, CH_3), 1.14 (dd, J = 29.6, 8.0 Hz, 3H, cyc-H), 0.87 (s, 2H, cyc-H). ^{13}C NMR (100 MHz, $CDCl_3$) δ = 157.9, 156.4, 156.0, 144.9, 133.0, 131.1, 129.9, 129.0, 128.4, 128.0, 114.6, 79.4, 70.8, 70.8, 69.8, 69.3, 68.8, 67.5, 52.5, 52.2, 50.5, 49.5, 36.9, 36.6, 31.0, 28.5, 26.6, 26.0, 21.7. HRMS (MALDI-TOF): $C_{32}H_{47}NO_8SNa$ [M+Na]$^+$: calcd 628.2925; found 628.2956.

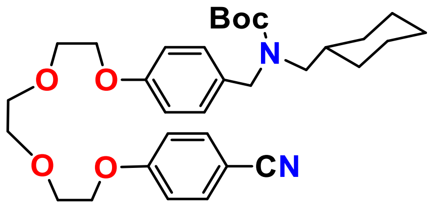

Synthesis of compound **6**

A solution of compound **5** (14.02 g, 23.1 mmol), 4-hydroxybenzonitrile (3.03 g, 25.5 mmol) and K_2CO_3 (10.55 g, 76.4 mmol) in $(CH_3)_2CO$ (150 mL) was heated at reflux for 24 h. The resultant mixture was cooled down and filtered with a pack of celite. The filtrate was evaporated to dryness and the residue was partitioned between NaOH (0.1 M, 100 mL) and EtOAc (50 mL). The aqueous layer was further extracted with EtOAc (3 × 50 mL). The combined organic layer was dried over anhydrous $MgSO_4$ and the filtrate was evaporated to dryness. Flash column chromatography with n-hex/EtOAc (3:1) on silica gel of the residue gave the compound **6** (9.21 g, 72 %) as a colorless liquid. R_f: 0.63 (n-hex/EtOAc = 3:1). ^{1}H NMR (400 MHz, $CDCl_3$) δ = 7.52 (d, *J* = 8.9 Hz, 2H, Ar*H*), 7.10 (s, 2H, Ar*H*), 6.92 (d, *J* = 8.9 Hz, 2H, Ar*H*), 6.82 (d, *J* = 8.6 Hz, 2H, Ar*H*), 4.33 (d, *J* = 19.6 Hz, 2H, C*H*$_2$NH), 4.17 – 4.11 (m, 2H, C*H*$_2$O–), 4.11 – 4.04 (m, 2H, C*H*$_2$O–), 3.88 – 3.79 (m, 4H, C*H*$_2$O–), 3.71 (s, 4H, C*H*$_2$O–), 2.96 (d, *J* = 30.7 Hz, 2H, C*H*$_2$NH), 1.64 (dd, *J* = 23.4, 10.6 Hz, 6H, cyc-*H*), 1.42 (d, *J* = 19.8 Hz, 9H, C*H*$_3$), 1.22 – 1.06 (m, 3H, cyc-*H*), 0.86 (s, 2H, cyc-*H*). ^{13}C NMR (100 MHz, $CDCl_3$) δ 162.1, 157.8, 156.3, 155.9, 133.9, 131.0, 130.8, 128.9, 128.3, 119.2, 115.3, 114.5, 104.0, 79.3, 70.9, 70.8, 69.8, 69.4, 67.8, 67.4, 52.4, 52.2, 50.4, 49.4, 36.8, 36.5, 30.9, 28.4, 26.5, 25.9. HRMS (MALDI-TOF): $C_{32}H_{44}N_2O_6Na$ $[M+Na]^+$: calcd 575.3102; found 575.3100.

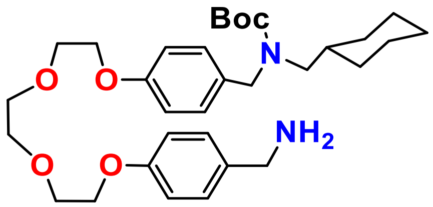

Synthesis of compound **7**

A solution of compound **6** (9.21 g, 16.7 mmol) in anhydrous THF (30 mL) was added dropwise to a suspension of $LiAlH_4$ (0.95 g, 25.0 mmol) in THF (50 mL) at 0 ºC. The resulting mixture was stirred at ambient temperature for 4 h. The resulting mixture was added to an ice bath and the suspension was filtered through a pack of celite. The filtrate was evaporated to dryness and the residue was partitioned between H_2O (100 mL) and $CHCl_3$ (50 mL). The aqueous layer was further extracted with $CHCl_3$ (3 × 50 mL). The combined organic layer was dried over anhydrous $MgSO_4$ and the filtrate was evaporated to dryness. Flash column chromatography with $CHCl_3$/EtOH (3:1) on silica gel of the residue gave the compound **7** (8.74 g, 94 %) as a pale-yellow liquid. R_f: 0.31 ($CHCl_3$/EtOH = 3:1). ^{1}H NMR (400 MHz, $CDCl_3$) δ 7.14 (d, J = 8.6 Hz, 2H, ArH), 7.08 (s, 2H, ArH), 6.81 (dd, J = 8.4, 5.9 Hz, 4H, ArH), 4.31 (d, J = 21.5 Hz, 2H, CH_2NH), 4.09 – 4.00 (m, 4H, CH_2O–), 3.83 – 3.75 (m, 4H, CH_2O–), 3.72 (s, 2H, CH_2NH), 3.68 (s, 4H, CH_2O–), 2.94 (d, J = 33.3 Hz, 2H, CH_2NH), 1.90 (s, 2H, NH_2), 1.62 (dd, J = 22.8, 9.7 Hz, 6H, cyc-H), 1.41 (d, J = 21.1 Hz, 9H, CH_3), 1.22 – 1.03 (m, 3H, cyc-H), 0.92 – 0.77 (m, 2H, cyc-H). ^{13}C NMR (100 MHz, $CDCl_3$) δ = 157.8, 157.6, 156.2, 155.8, 135.4, 130.8, 130.6, 128.8, 128.1, 114.5, 114.4, 79.2, 70.7, 69.7, 67.3, 52.3, 52.0, 50.3, 49.3, 45.6, 36.7, 36.4, 30.8, 28.3, 26.4, 25.8. HRMS (MALDI-TOF): $C_{32}H_{48}N_2O_6Na$ $[M+Na]^+$: calcd 579.3404; found 579.3442.

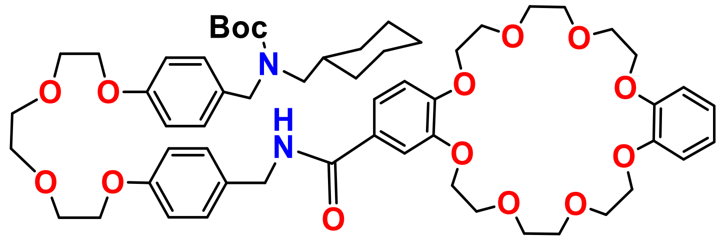

Synthesis of compound **8**

A solution of compound **7** (3.17 g, 5.69 mmol) and dibenzo[24]crown-8-OSu (3.36 g, 5.69 mmol) in CH_2Cl_2 (30 mL) was heated at reflux for 12 h. The resulting solution was evaporated to dryness and the residue was partitioned between H_2O (50 mL) and $CHCl_3$ (25 mL). The aqueous layer was further extracted with $CHCl_3$ (3 × 25 mL). The combined organic layer was dried over anhydrous $MgSO_4$ and the filtrate was evaporated to dryness. Flash column chromatography with EtOAc on silica gel of the residue gave the compound **8** (4.35 g, 74 %) as a white powder. M.P.: 98.7 – 99.4 ºC. *Rf*: 0.43 (EtOAc). ^{1}H NMR (400 MHz, CD_2Cl_2) δ = 7.42 (d, *J* = 1.8 Hz, 1H, Ar*H*), 7.36 (s, 1H, Ar*H*), 7.25 (d, *J* = 8.7 Hz, 2H, Ar*H*), 7.14 (d, *J* = 8.6 Hz, 2H, Ar*H*), 6.94 – 6.81 (m, 10H, Ar*H* & –CON*H*–), 4.49 (d, *J* = 5.7 Hz, 2H, –CONHC*H*2), 4.35 (s, 2H, C*H*2NH), 4.15 – 4.06 (m, 12H, C*H*2O–), 3.88 – 3.79 (m, 12H, C*H*2O–), 3.76 (d, *J* = 4.9 Hz, 8H, C*H*2O–), 3.69 (s, 4H, C*H*2O–), 2.98 (d, *J* = 11.1 Hz, 2H, C*H*2NH), 1.67 (dd, *J* = 27.4, 12.1 Hz, 6H, cyc-*H*), 1.44 (d, *J* = 20.3 Hz, 9H, C*H*3), 1.28 – 1.12 (m, 3H, cyc-*H*), 0.96 – 0.83 (m, 2H, cyc-*H*). ^{13}C NMR (101 MHz, CD_2Cl_2) δ = 166.8, 158.4, 158.2, 156.4, 156.0, 151.9, 149.30, 149.28, 148.8, 131.6, 131.3, 129.4, 129.1, 128.8, 127.8, 121.69, 121.67, 120.5, 114.80, 114.6, 114.48, 114.45, 113.3, 112.7, 79.4, 78.0, 71.5, 71.4, 71.1, 70.1, 70.02, 70.00, 69.9, 69.6, 69.52, 69.47, 69.45, 52.6, 50.5, 49.7, 43.6, 37.2, 36.9, 31.3, 28.5, 26.9, 26.3. HRMS (MALDI-TOF): $C_{57}H_{78}N_2O_{15}Na$ $[M+Na]^+$: calcd 1053.5294; found 1053.5307.

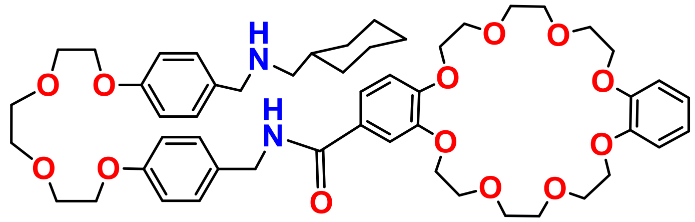

Synthesis of compound **9**

A solution of compound **8** (4.35 g, 4.21 mmol) and TFA (5 mL) in CH_2Cl_2 (30 mL) was stirred at ambient temperature for 6 h. The resulting solution was neutralized with sat. Na_2CO_3 solution and washed with H_2O (3 × 15 mL). The organic layer was dried over anhydrous $MgSO_4$ and the filtrate was evaporated to give compound **9** (3.68 g, 94 %) as a white powder. M.P.: 117.3 – 118.7 ºC. R_f: 0.11 (EtOAc). ^{1}H NMR (400 MHz, CD_2Cl_2) δ = 7.38 (d, *J* = 2.0 Hz, 1H, Ar*H*), 7.29 (dd, *J* = 8.4, 2.1 Hz, 1H, Ar*H*), 7.25 (d, *J* = 8.6 Hz, 2H, Ar*H*), 7.21 (d, *J* = 8.5 Hz, 2H, Ar*H*), 6.91 – 6.81 (m, 9H, Ar*H*), 6.50 (t, *J* = 5.6 Hz, 1H, –CON*H*–), 4.50 (d, *J* = 5.7 Hz, 2H, –CONHCH_2), 4.18 – 4.12 (m, 4H, CH_2O–), 4.12 – 4.06 (m, 8H, CH_2O–), 3.85 (dt, *J* = 8.9, 5.1 Hz, 8H, CH_2O–), 3.83 – 3.79 (m, 4H, CH_2O–), 3.76 (d, *J* = 3.1 Hz, 8H, CH_2O–), 3.69 (s, 4H, CH_2O–), 3.66 (s, 2H, CH_2NH), 2.41 (d, *J* = 6.7 Hz, 2H, CH_2NH), 1.76 – 1.62 (m, 6H, cyc-*H* & –N*H*), 1.43 (ddd, *J* = 11.2, 6.9, 3.4 Hz, 1H, cyc-*H*), 1.28 – 1.11 (m, 3H, cyc-*H*), 0.95 – 0.84 (m, 2H, cyc-*H*). ^{13}C NMR (100 MHz, CD_2Cl_2) δ = 166.8, 158.5, 158.1, 152.0, 149.37, 149.35, 149.0, 131.4, 129.6, 129.4, 127.8, 121.70, 121.67, 120.3, 114.9, 114.6, 114.53, 114.48, 113.3, 112.8, 71.6, 71.5, 71.4, 71.13, 71.12, 70.20, 70.19, 70.11, 70.09, 70.05, 70.00, 69.8, 69.6, 69.5, 67.9, 67.8, 56.5, 53.7, 43.7, 38.4, 31.9, 27.1, 26.5. HRMS (MALDI-TOF): $C_{52}H_{70}N_2O_{13}Na$ $[M+Na]^+$: calcd 953.4770; found 953.4757.

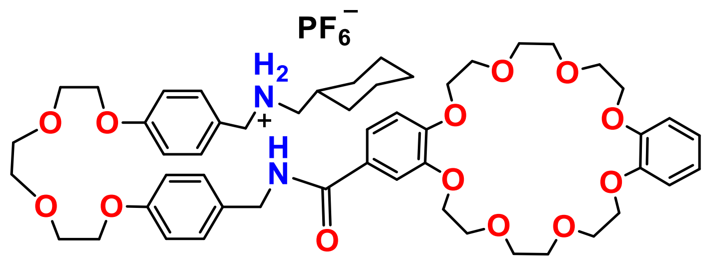

Synthesis of **1-H·PF$_6$**

A solution of compound **9** (3.68 g, 4.0 mmol) in 30 mL CH_2Cl_2/CH_3OH (5:1) was stirred at 0 ºC. The solution was acidified with conc. HCl until the pH of the solution reached pH 3 and the resulting solution was stirred at ambient temperature for 2 h. The solution was dried under reduced pressure, and the residue was re-dissolved in $(CH_3)_2CO$ (30 mL). A solution of sat. NH_4PF_6 (3 mL) was added to the reaction mixture and the resulting solution was stirred at ambient temperature for another 2 h. The reaction mixture was evaporated to dryness, and the residue was partitioned between H_2O (30 mL) and $CHCl_3$ (15 mL). The aqueous layer was further extracted with $CHCl_3$ (3 × 15 mL). The combined organic layer was dried over anhydrous $MgSO_4$ and the filtrate was dried at reduced pressure to give **1-H·PF$_6$** (3.45 g, 81 %) as a white glassy powder. M.P.: 74.2 – 75.7 ºC. R_f: 0.61 (DCM/MeCN = 1:1). 1H NMR (400 MHz, CD_2Cl_2) δ = 7.37 (dd, J = 8.4, 1.5 Hz, 1H, Ar*H*), 7.32 (s, 1H, Ar*H*), 7.19 (d, J = 8.7 Hz, 2H, Ar*H*), 7.15 (d, J = 5.8 Hz, 1H, Ar*H*), 7.11 (d, J = 8.7 Hz, 2H, Ar*H*), 7.00 – 6.91 (m, 5H, Ar*H* & –CON*H*–), 6.88 (d, J = 8.7 Hz, 2H, Ar*H*), 6.74 (d, J = 8.7 Hz, 2H, Ar*H*), 4.42 (d, J = 5.9 Hz, 2H, –CONHCH_2), 4.24 – 4.12 (m, 8H, CH_2O–), 4.09 (dd, J = 5.4, 3.6 Hz, 2H, CH_2NH), 4.03 – 3.96 (m, 4H, CH_2O–), 3.90 – 3.81 (m, 8H, CH_2O–), 3.81 – 3.70 (m, 12H, CH_2O–), 3.65 (d, J = 2.2 Hz, 4H, CH_2O–), 2.65 (d, J = 6.8 Hz, 2H, CH_2NH), 1.63 (d, J = 10.1 Hz, 8H, cyc-*H* & –N*H*), 1.10 (t, J = 9.5 Hz, 3H, cyc-*H*), 0.91 – 0.80 (m, 2H, cyc-*H*). ^{13}C NMR (100 MHz, CD_2Cl_2) δ = 167.8, 160.2, 158.4, 151.5, 148.7, 148.5, 148.3, 132.1, 131.3, 129.2, 123.2, 123.1, 122.3, 122.0, 116.0, 115.5, 115.0, 71.0, 70.0, 69.9, 69.8, 69.7, 69.4, 69.3, 69.2, 68.9, 68.8, 68.02,

67.99, 52.4, 43.8, 35.2, 30.5, 26.1, 25.7. HRMS (MALDI-TOF): $C_{52}H_{70}N_2O_{13}Na$ $[M+Na]^+$: calcd 953.4770; found 953.4751.

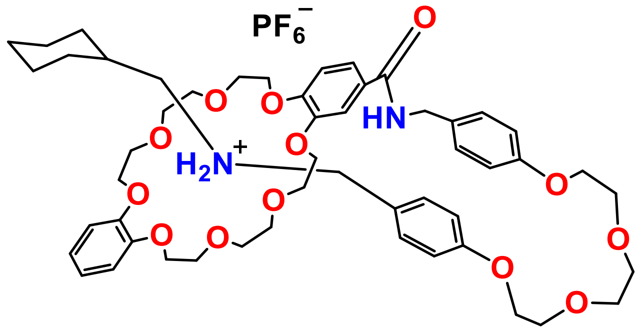

Synthesis of **1-H(Rot)·PF$_6$**

A solution of compound **1-H·PF$_6$** (0.21 g, 0.20 mmol) and KPF_6 (0.036 g, 0.20 mmol) in CH_3CN (1 mL) was heated at 65 ºC in Schlenk flask for 24 h.[3] The resulting solution was evaporated to dryness, and the residue was partitioned between H_2O (5 mL) and CH_2Cl_2 (2.5 mL). The aqueous layer was further extracted by CH_2Cl_2 (3 × 2.5 mL). The combined organic layer was dried over anhydrous $MgSO_4$, and the filtrate was evaporated to dryness. Flash column chromatography with CH_2Cl_2/CH_3CN (1:1) on silica gel of the residue gave the compound **1-H(Rot)·PF$_6$** (0.065 g, 31 %) as a white paste. R_f: 0.58. ^{1}H NMR (400 MHz, CD_2Cl_2) δ = 7.57 (d, J = 2.0 Hz, 1H, Ar*H*), 7.49 (dd, J = 8.5, 2.0 Hz, 1H, Ar*H*), 7.38 (t, J = 6.0 Hz, 1H, –CON*H*–), 7.24 (d, J = 8.7 Hz, 2H, Ar*H*), 7.21 (d, J = 8.7 Hz, 2H, Ar*H*), 7.15 (d, J = 8.6 Hz, 1H, Ar*H*), 7.12 – 7.05 (m, 4H, Ar*H*), 6.82 (dd, J = 11.7, 8.7 Hz, 4H, Ar*H*), 4.42 (d, J = 5.8 Hz, 2H, –CONHC*H*$_2$), 4.38 (dd, J = 5.3, 3.3 Hz, 2H, C*H*$_2$O–), 4.35 (dd, J = 5.3, 3.5 Hz, 2H, C*H*$_2$O–), 4.29 (dd, J = 9.1, 6.5 Hz, 4H, C*H*$_2$O–), 4.09 – 4.01 (m, 6H, C*H*$_2$O– & C*H*$_2$NH), 3.79 (dd, J = 5.4, 2.9 Hz, 8H, C*H*$_2$O–), 3.73 (dd, J = 8.7, 4.9 Hz, 4H, C*H*$_2$O–), 3.67 (s, 4H, C*H*$_2$O–), 3.59 (s, 8H, C*H*$_2$O–), 2.75 (d, J = 6.5 Hz, 2H, C*H*$_2$NH), 2.15 (s, 2H, –N*H*), 1.66 – 1.54 (m, 6H, cyc-*H*), 1.10 (t, J = 9.7 Hz, 3H, cyc-*H*), 0.92 – 0.81 (m, 2H, cyc-*H*). ^{13}C NMR (100 MHz, CD_2Cl_2) δ = 167.7, 160.1, 158.2, 151.5, 148.5, 148.3, 148.2, 132.1, 131.4, 129.3, 129.1, 123.9,

123.8, 122.9, 122.4, 117.4, 116.7, 116.4, 116.3, 115.5, 115.0, 70.9, 70.0, 69.9, 69.5, 69.2, 68.6, 68.5, 68.0, 67.9, 67.8, 67.7, 67.6, 67.53 (s), 67.45 (s), 67.3, 67.2, 53.8, 52.4, 43.7, 35.3, 30.4, 26.1, 25.6. HRMS (MALDI-TOF): $C_{52}H_{70}N_2O_{13}Na$ $[M+Na]^+$: calcd 953.4770; found 953.4765.

3. NMR spectra of selected compounds

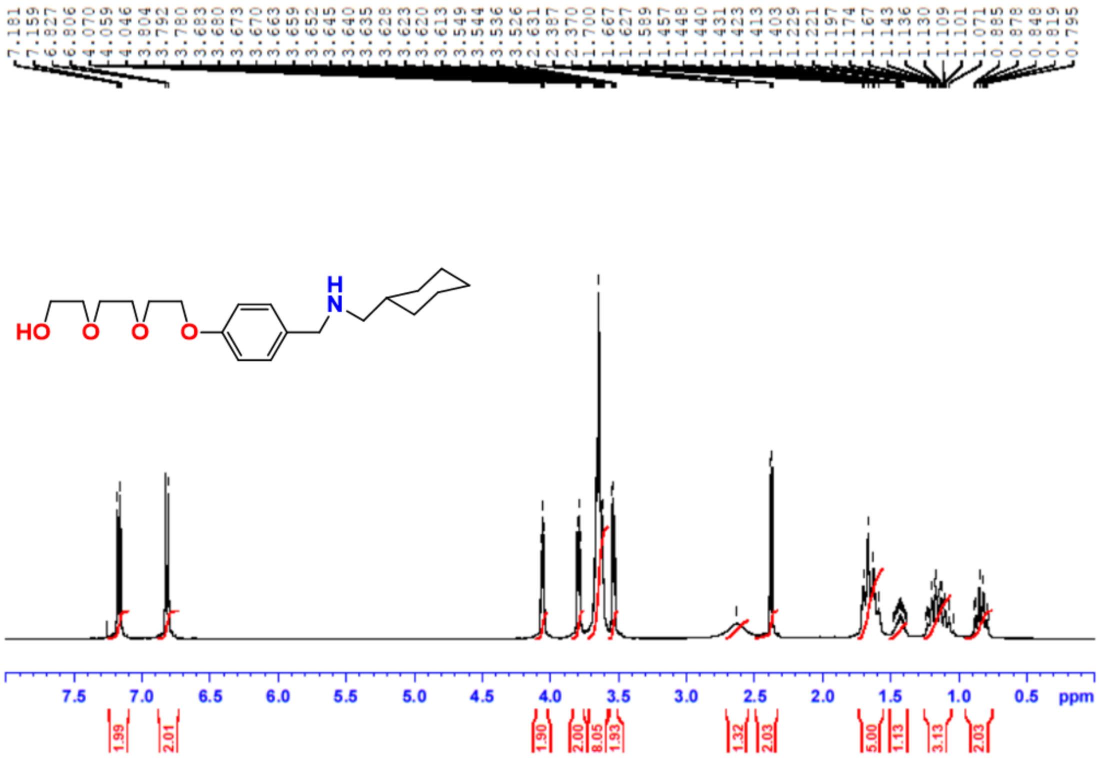

Figure S1. ^{1}H NMR spectrum (400 MHz, $CDCl_3$, 298 K) of compound **3**.

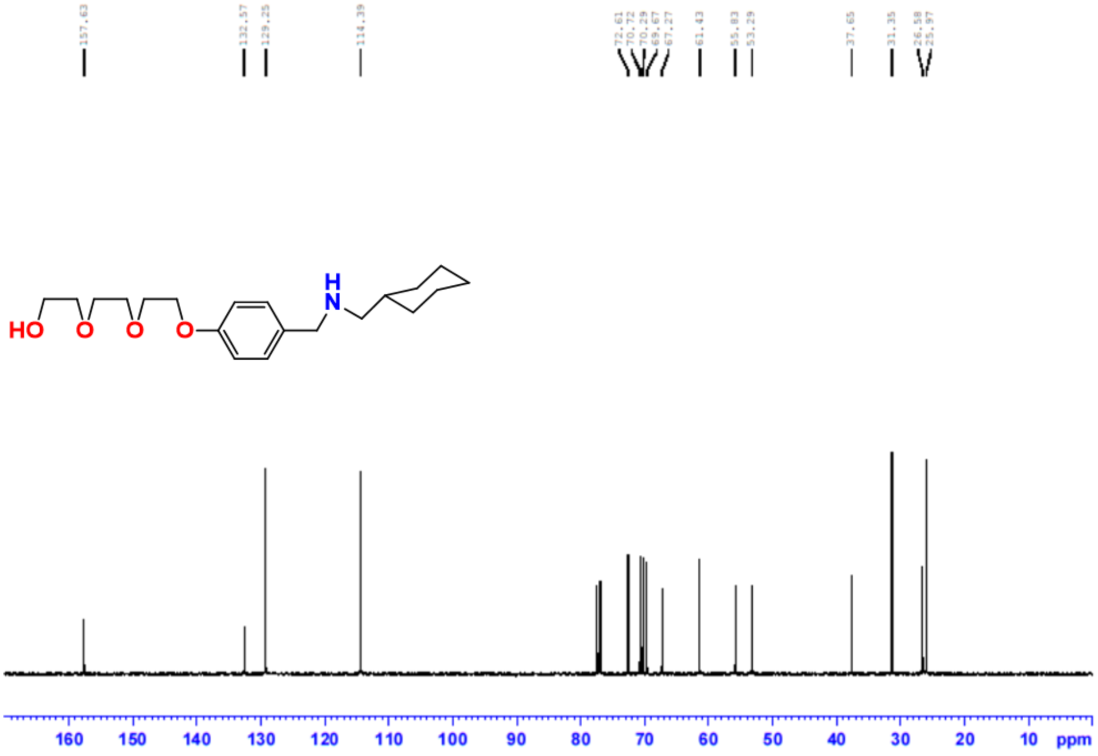

Figure S2. $^{13}C\{^{1}H\}$ NMR spectrum (101 MHz, $CDCl_3$, 298 K) of compound **3**.

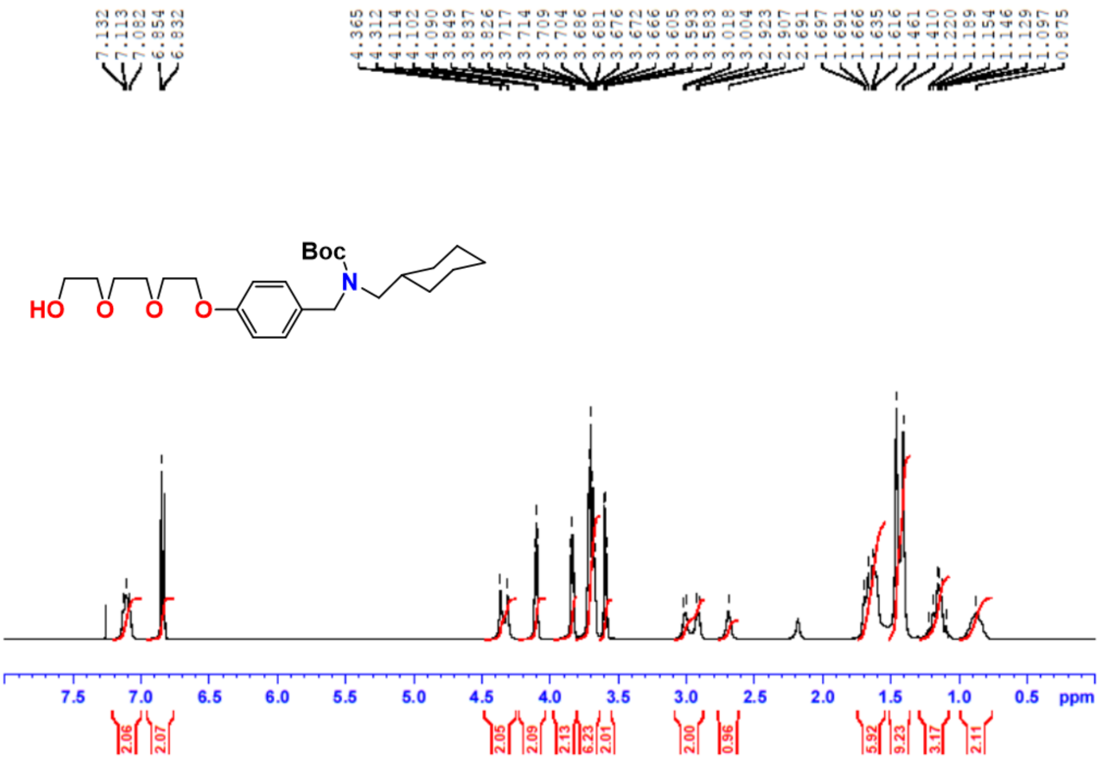

Figure S3. ^{1}H NMR spectrum (400 MHz, $CDCl_3$, 298 K) of compound **4**.

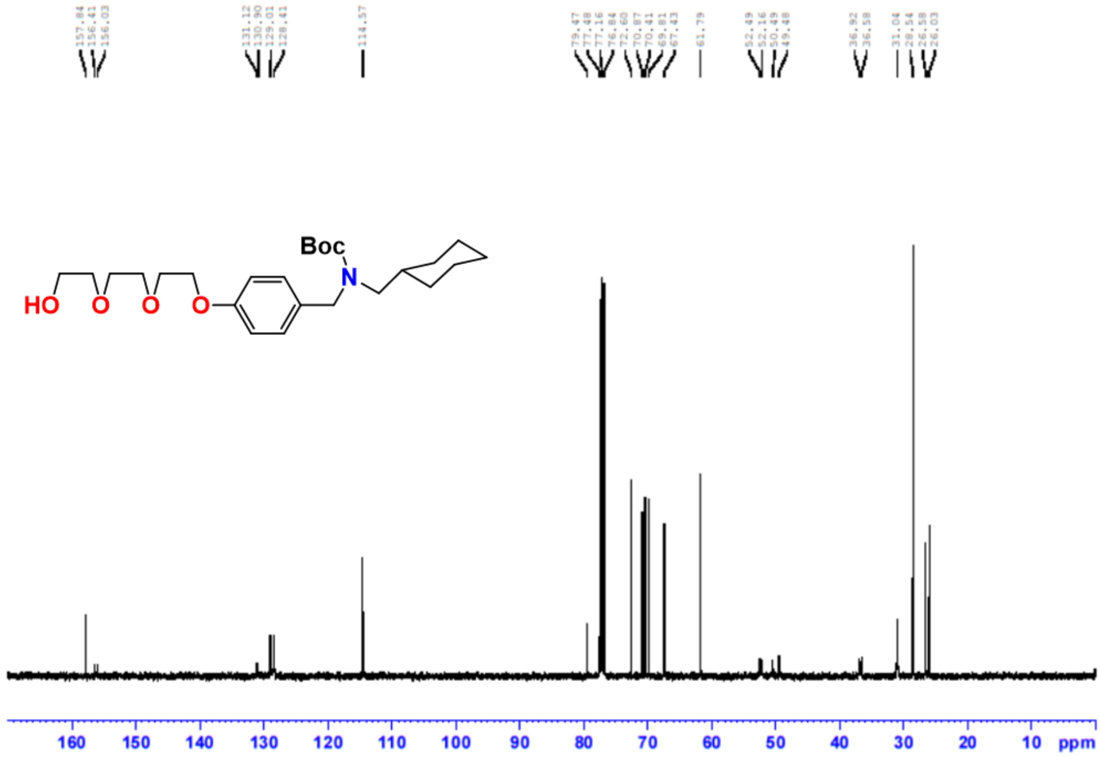

Figure S4. $^{13}C\{^{1}H\}$ NMR spectrum (101 MHz, $CDCl_3$, 298 K) of compound **4**.

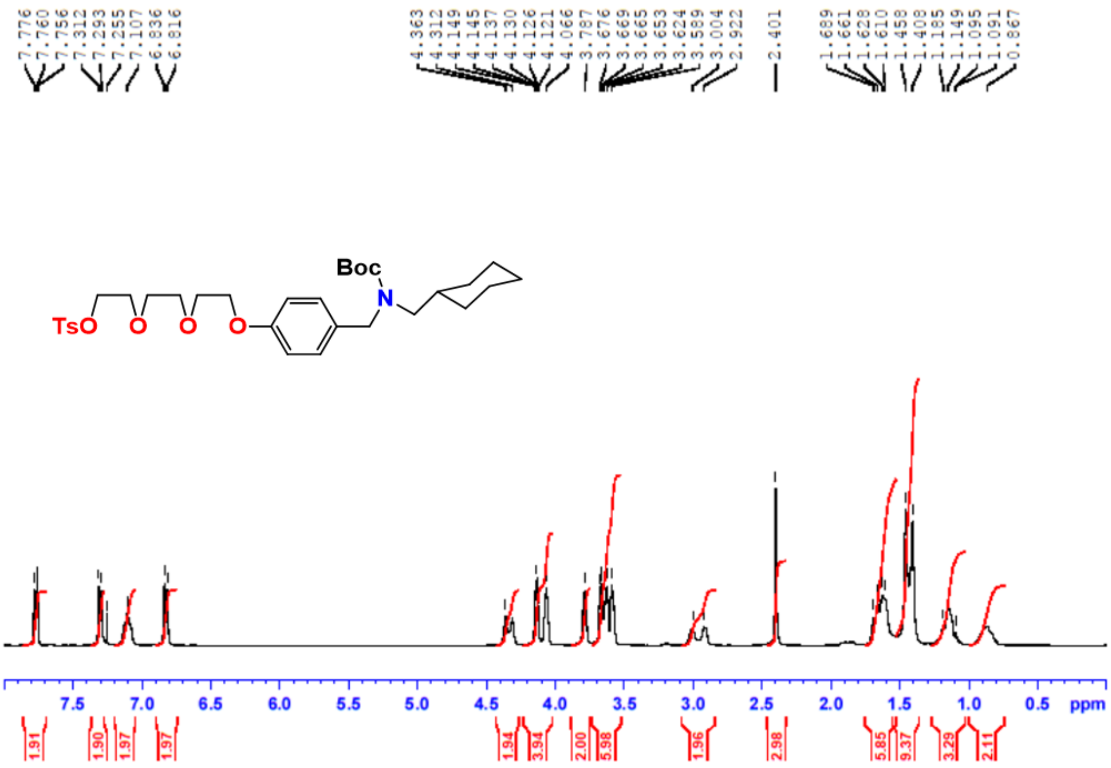

Figure S5. ^{1}H NMR spectrum (400 MHz, $CDCl_3$, 298 K) of compound **5**.

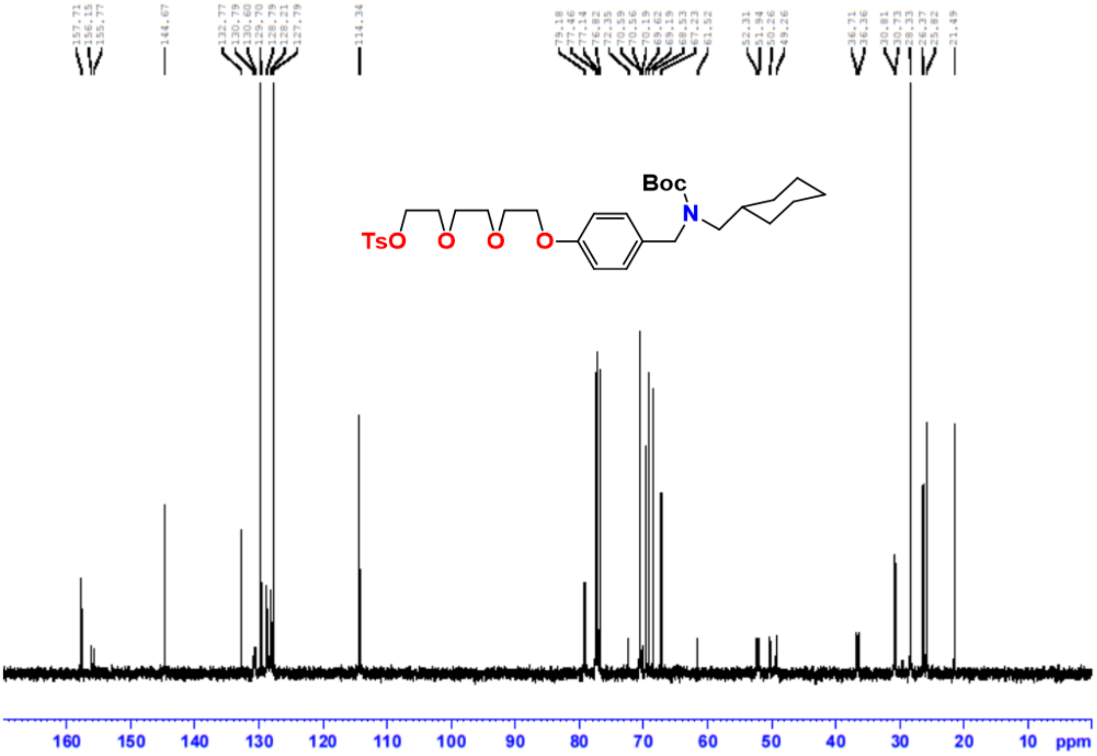

Figure S6. $^{13}C\{^{1}H\}$ NMR spectrum (101 MHz, $CDCl_3$, 298 K) of compound **5**.

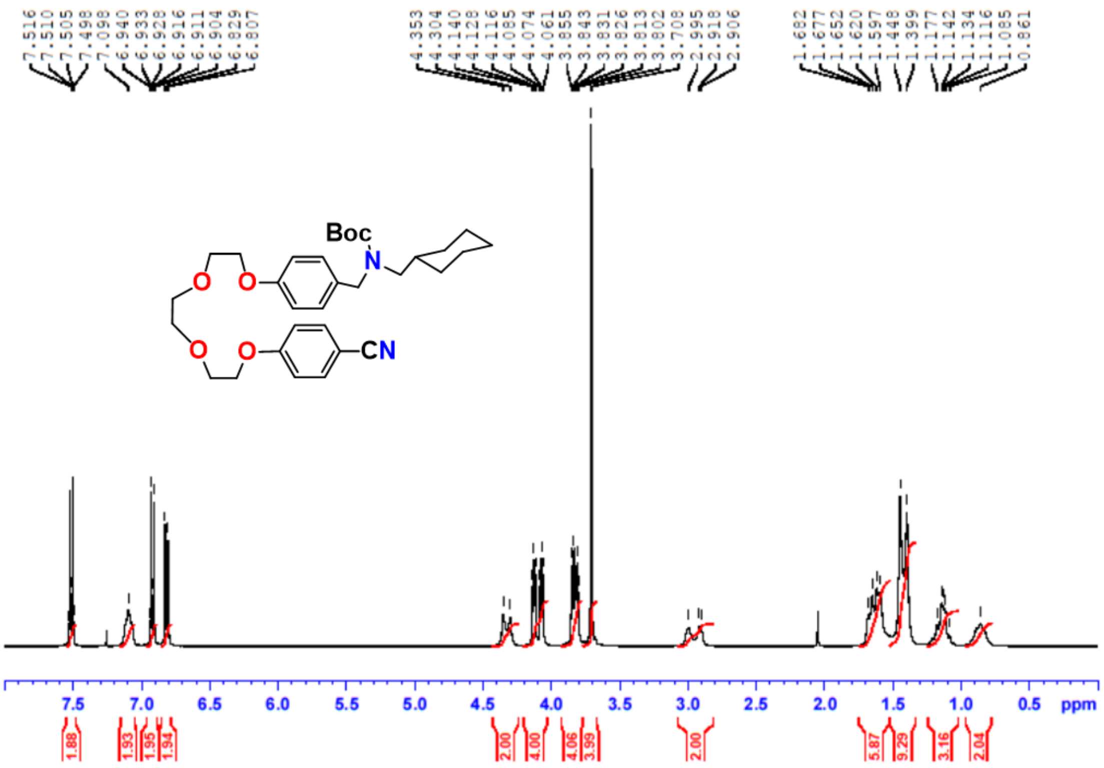

Figure S7. ^{1}H NMR spectrum (400 MHz, $CDCl_3$, 298 K) of compound **6**.

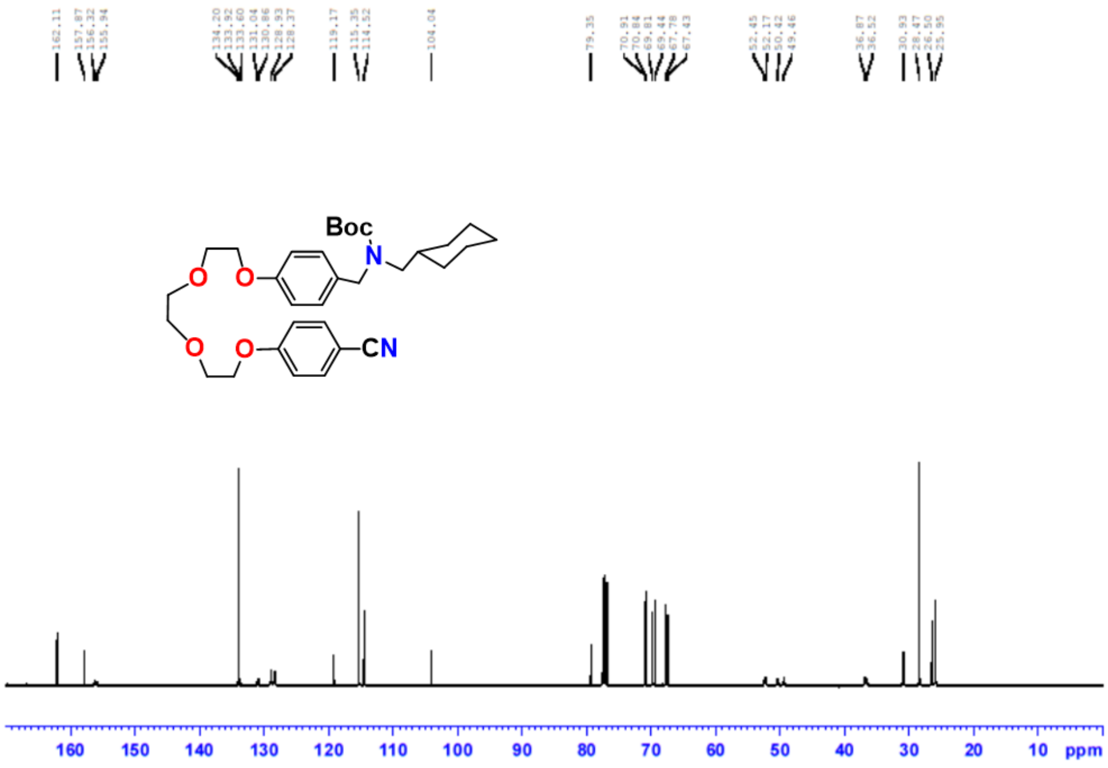

Figure S8. $^{13}C\{^1H\}$ NMR spectrum (101 MHz, $CDCl_3$, 298 K) of compound **6**.

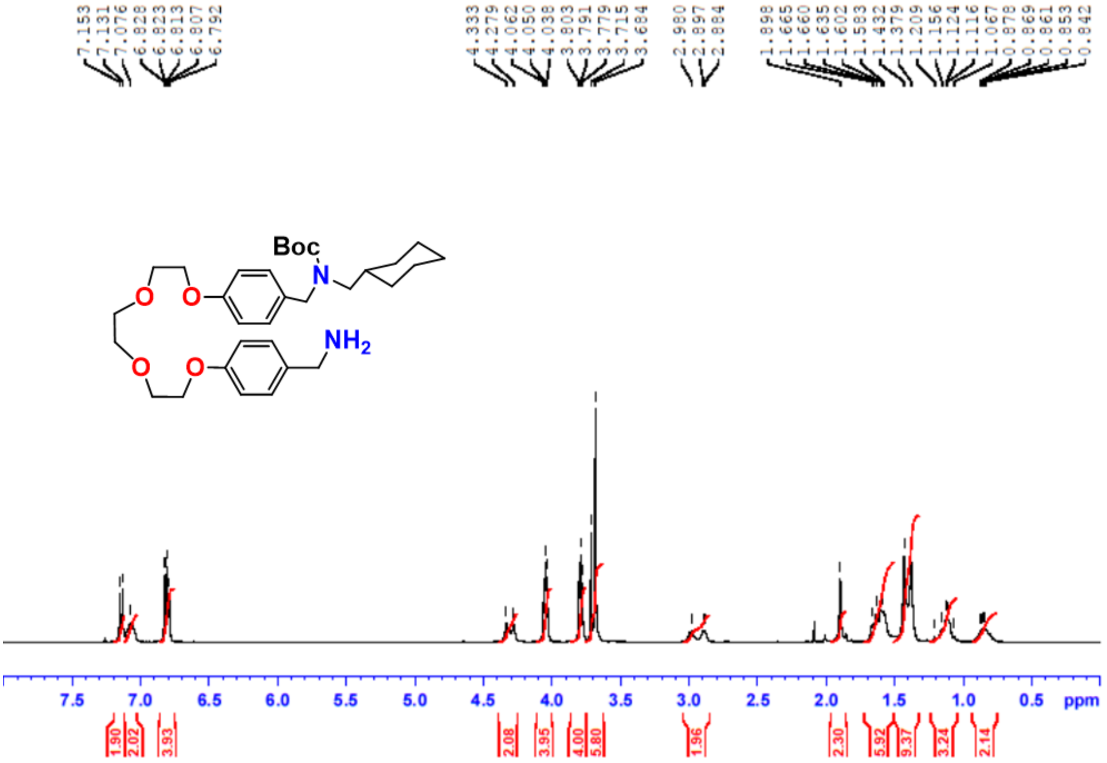

Figure S9. ^{1}H NMR spectrum (400 MHz, $CDCl_3$, 298 K) of compound **7**.

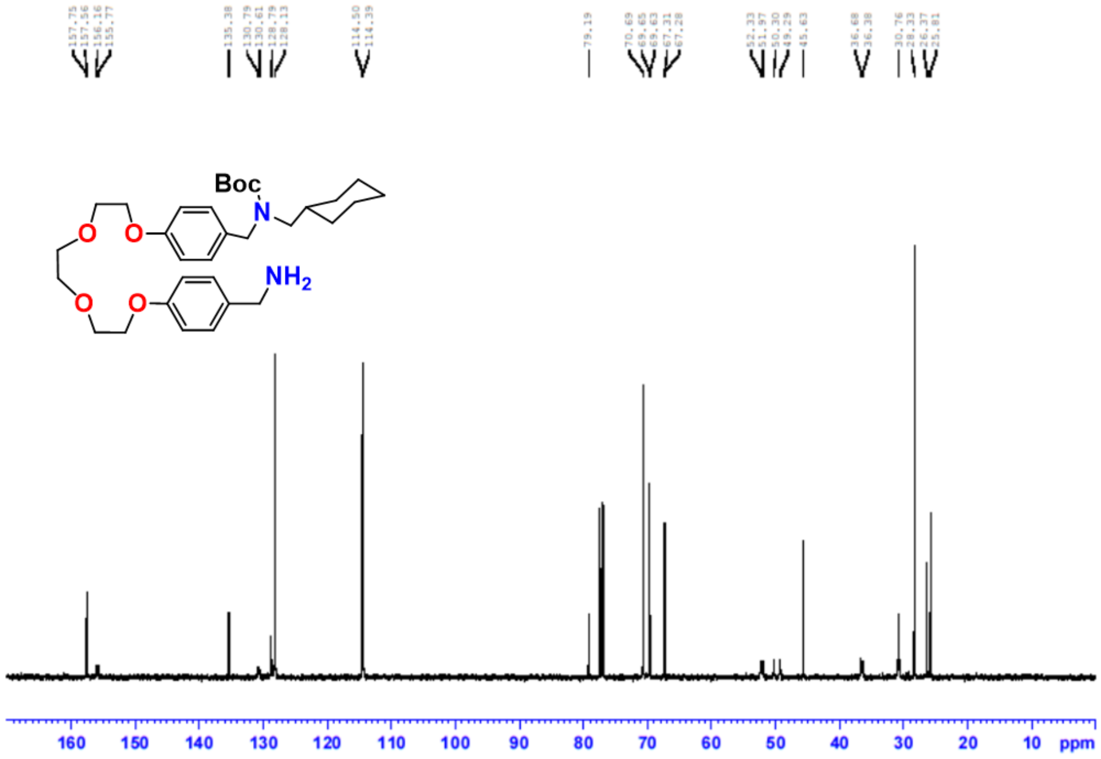

Figure S10. $^{13}C\{^{1}H\}$ NMR spectrum (101 MHz, $CDCl_3$, 298 K) of compound **7**.

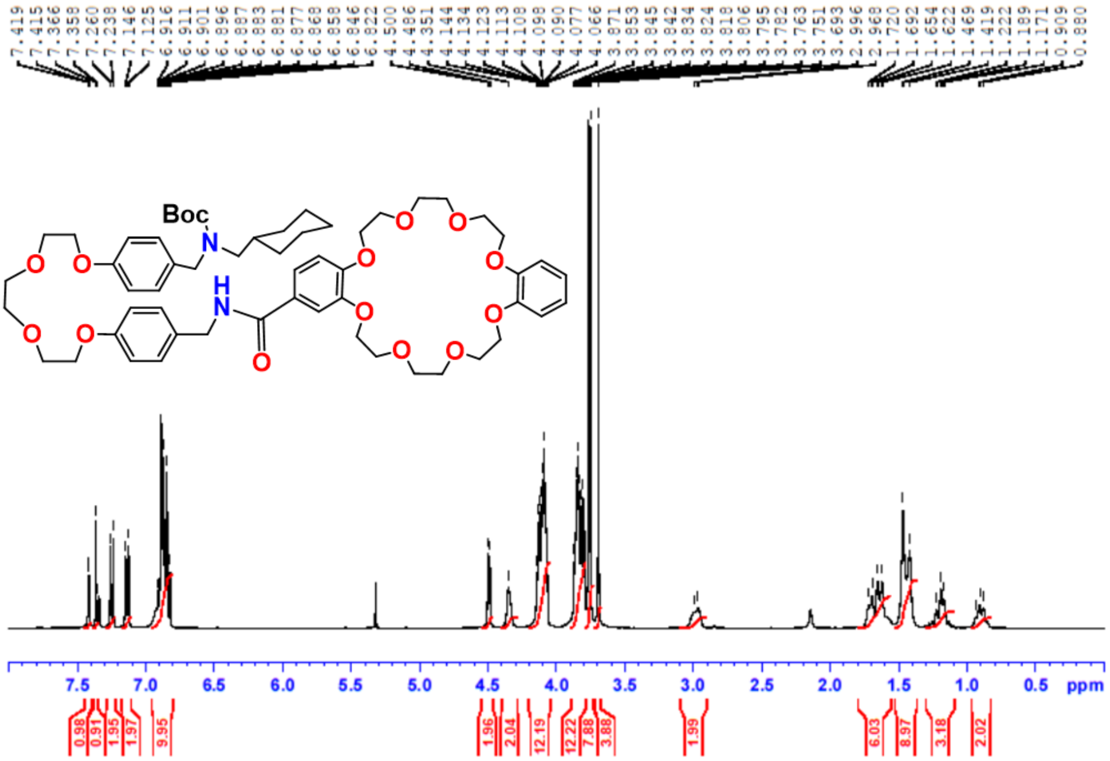

Figure S11. ^{1}H NMR spectrum (400 MHz, CD_2Cl_2, 298 K) of compound **8**.

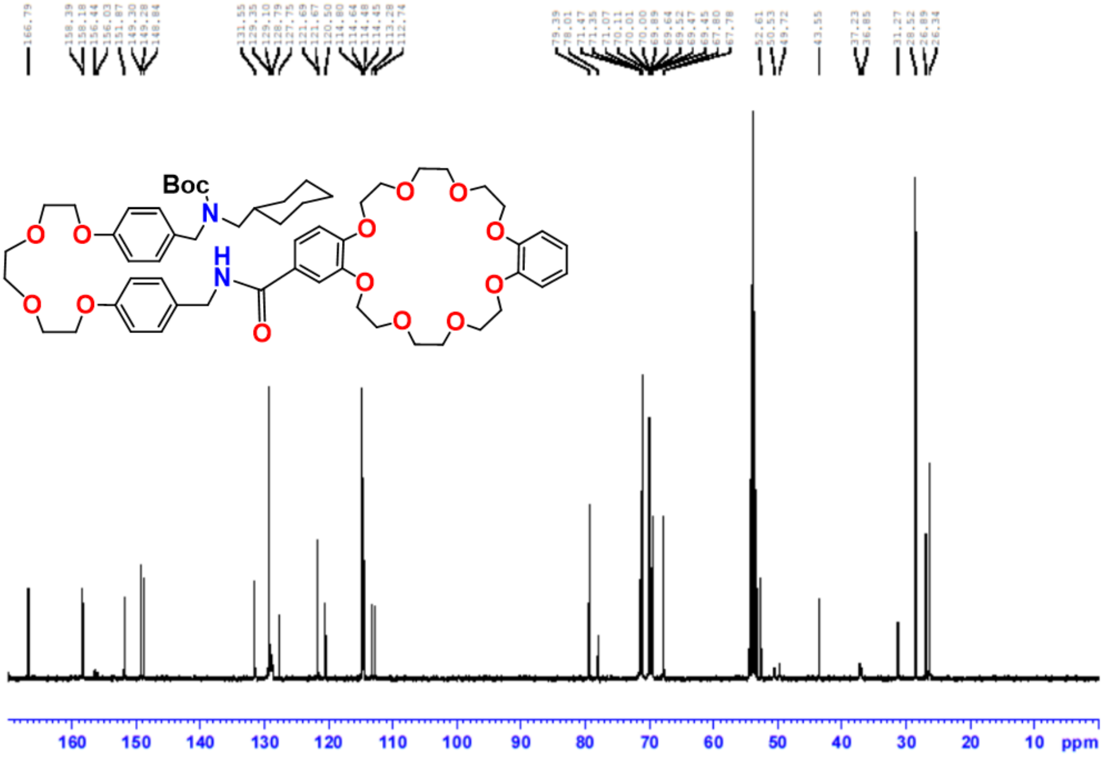

Figure S12. $^{13}C\{^{1}H\}$ NMR spectrum (101 MHz, CD_2Cl_2, 298 K) of compound **8**.

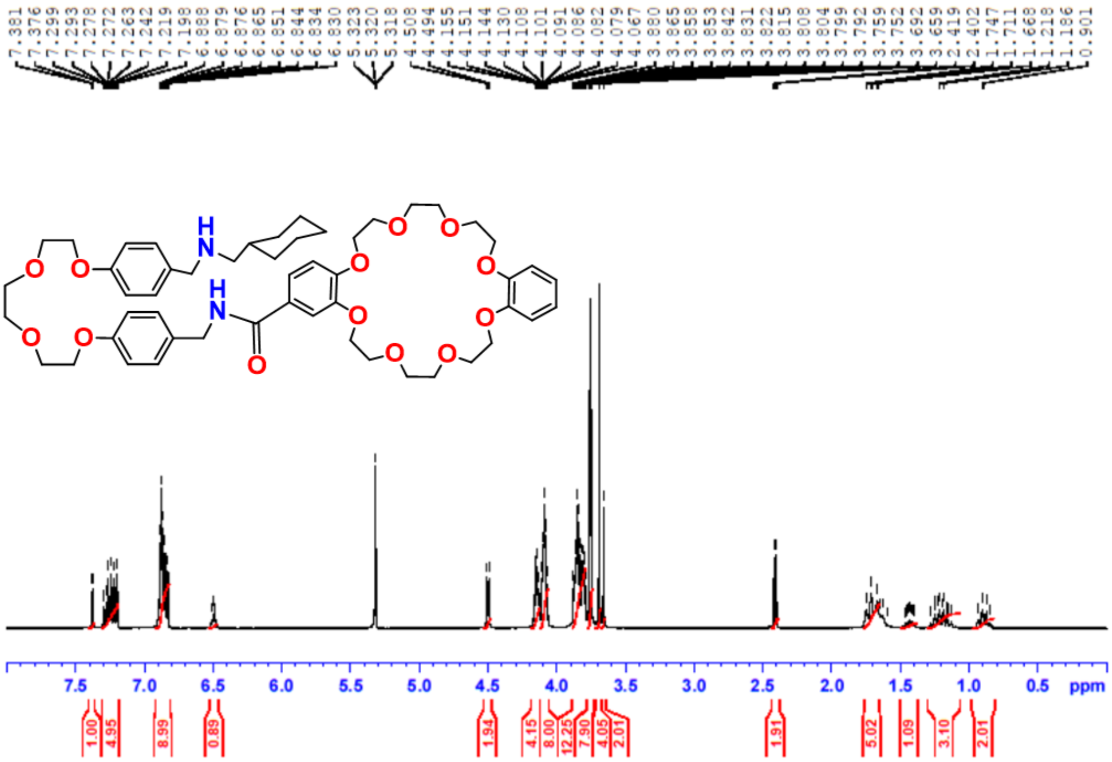

Figure S13. ^{1}H NMR spectrum (400 MHz, CD_2Cl_2, 298 K) of compound **9**.

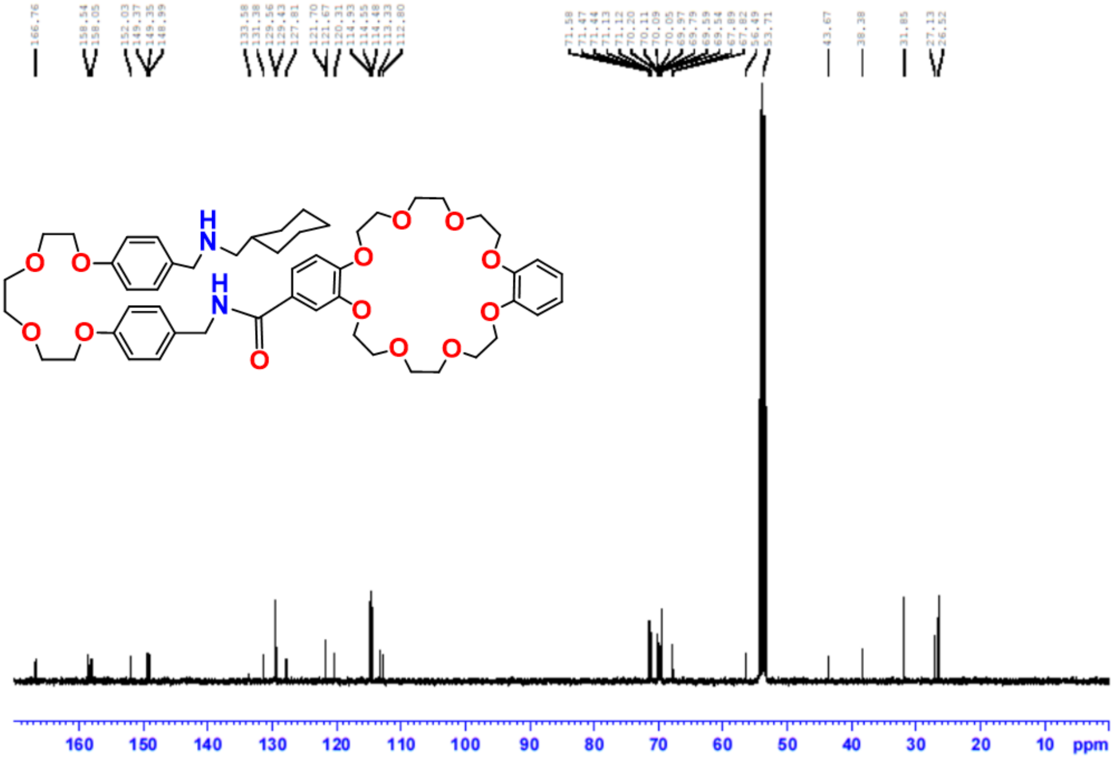

Figure S14. $^{13}C\{^{1}H\}$ NMR spectrum (101 MHz, CD_2Cl_2, 298 K) of compound **9**.

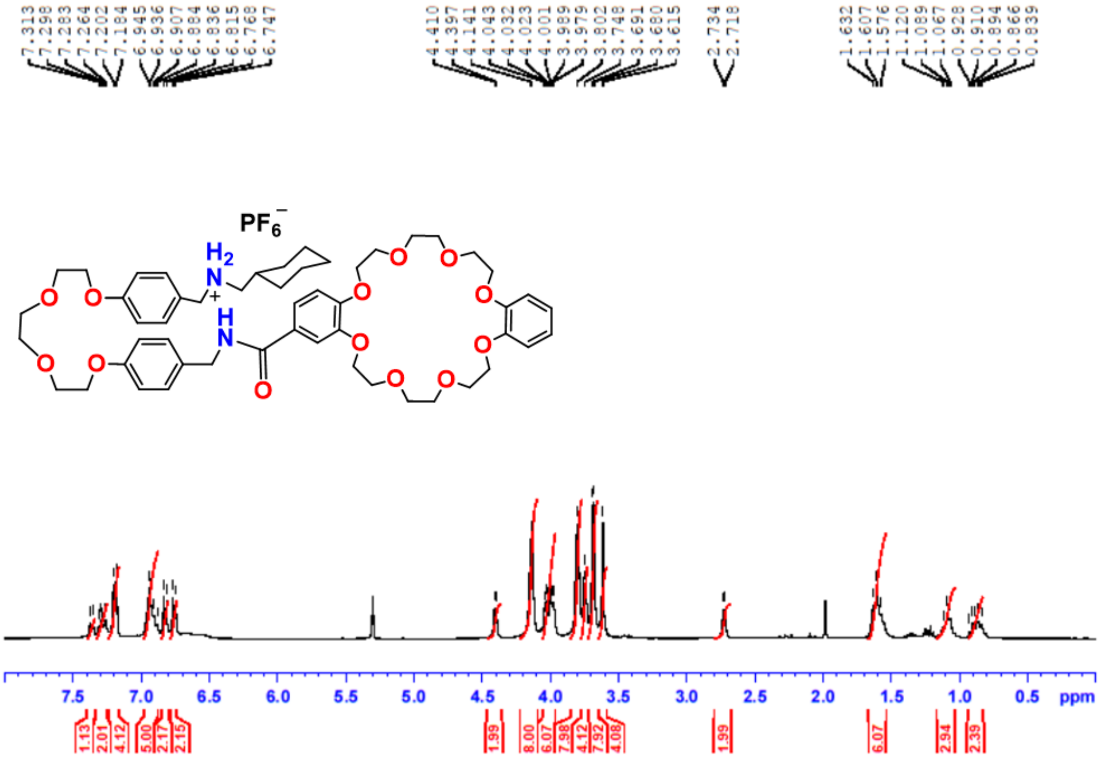

Figure S15. ^{1}H NMR spectrum (400 MHz, CD_2Cl_2, 298 K) of compound **1-H·PF$_6$**.

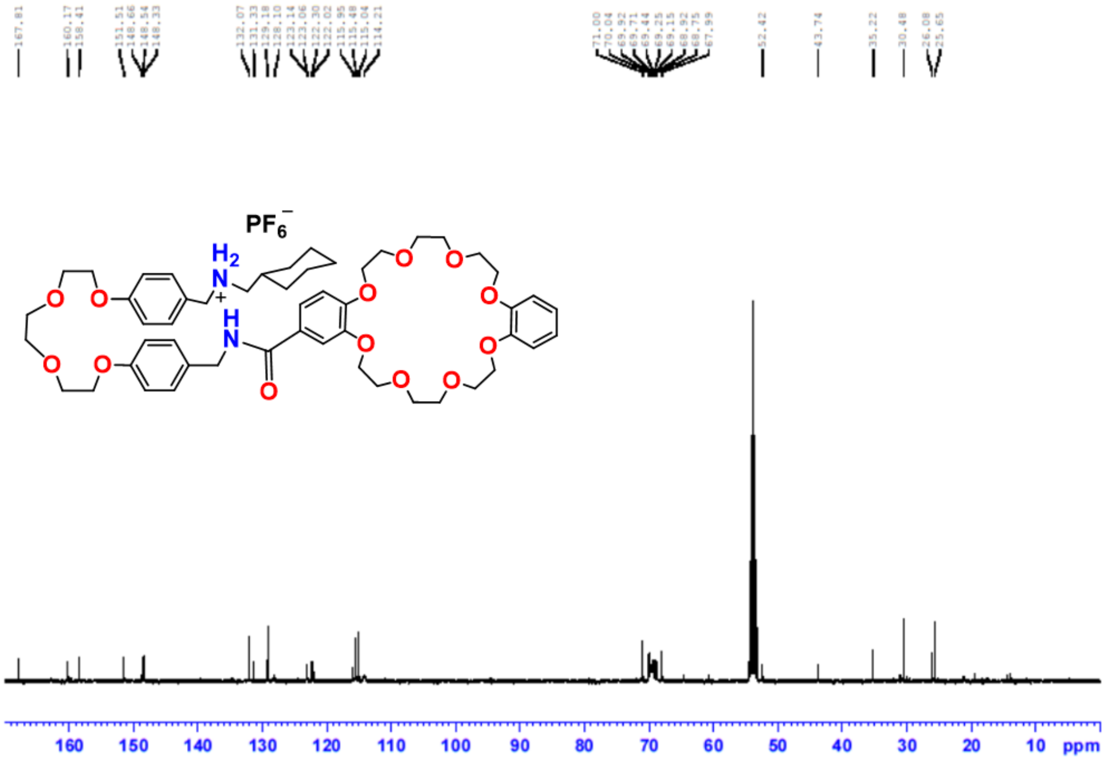

Figure S16. $^{13}C\{^{1}H\}$ NMR spectrum (101 MHz, CD_2Cl_2, 298 K) of compound **1-H·PF$_6$**.

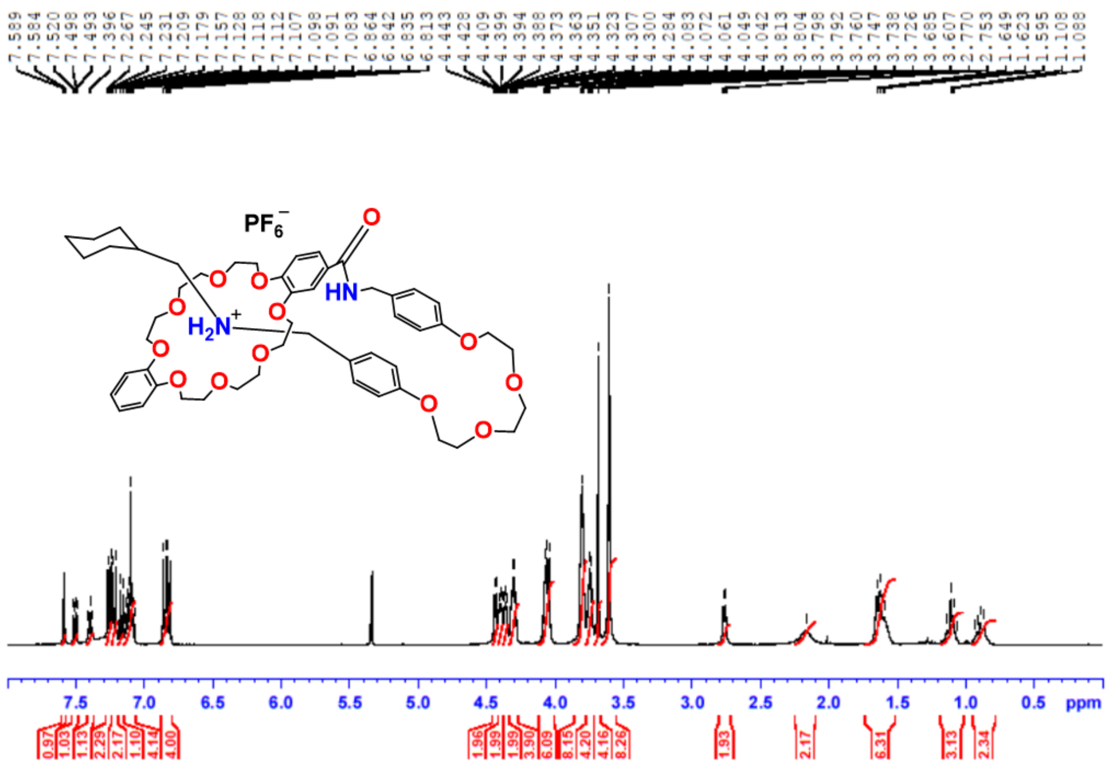

Figure S17. ^{1}H NMR spectrum (400 MHz, CD_2Cl_2, 298 K) of compound **1-H(Rot)·PF$_6$**.

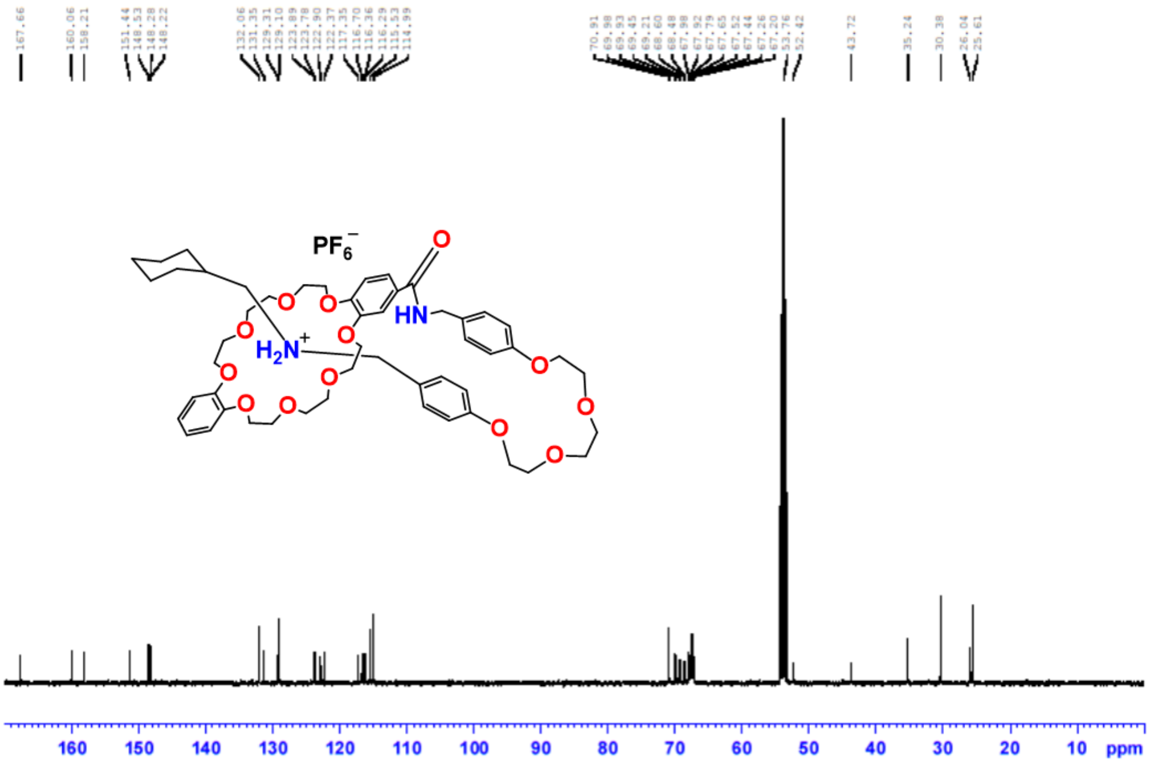

Figure S18. $^{13}C\{^{1}H\}$ NMR spectrum (101 MHz, CD_2Cl_2, 298 K) of compound **1-H·PF$_6$**.

Stacked ^{13}C NMR spectra of **1-H·PF$_6$** and **1-H(Rot)·PF$_6$** are shown in **Figure S19** with sharp signals. The aromatic carbons and ethylene carbons of **1-H(Rot)·PF$_6$** showed a new pattern because of the differences in chemical environment compared with **1-H·PF$_6$**, revealing the successful synthesis of the [1]rotaxane.

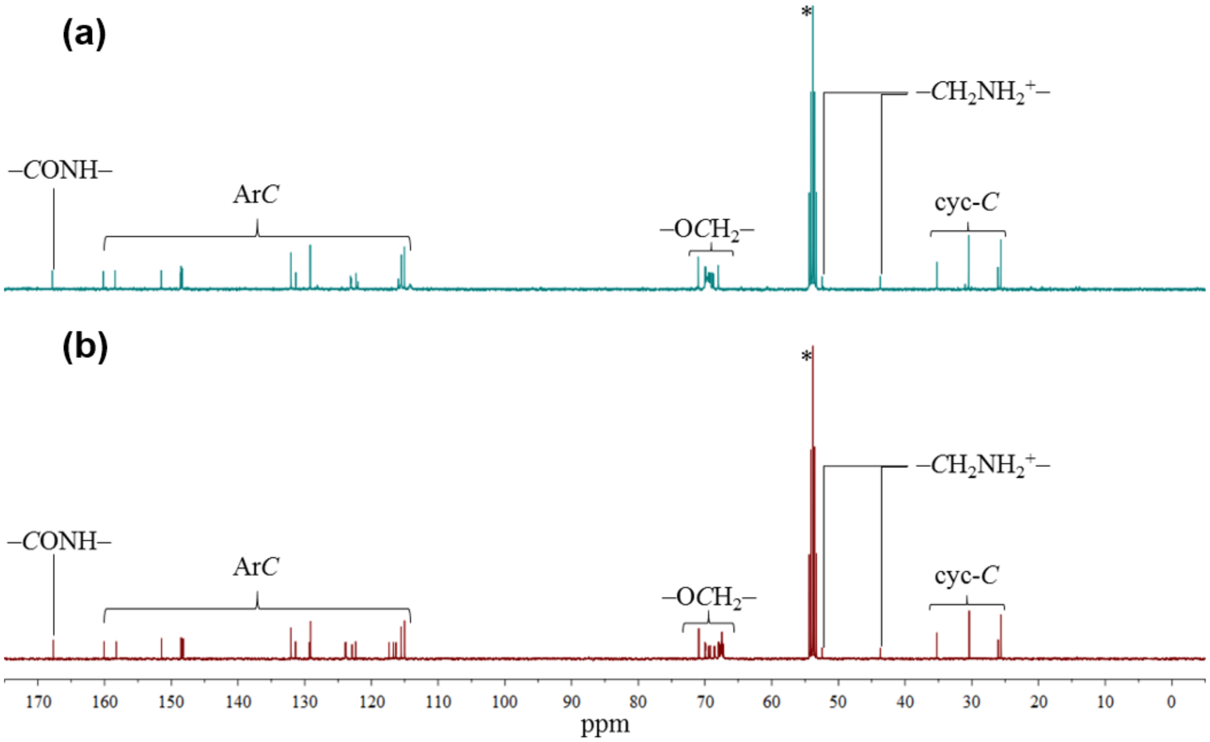

Figure S19. ^{13}C NMR (CD_2Cl_2, 298 K) spectra of compound (a) **1-H·PF$_6$** and (b) **1-H(Rot)·PF$_6$**. *: solvent residue.

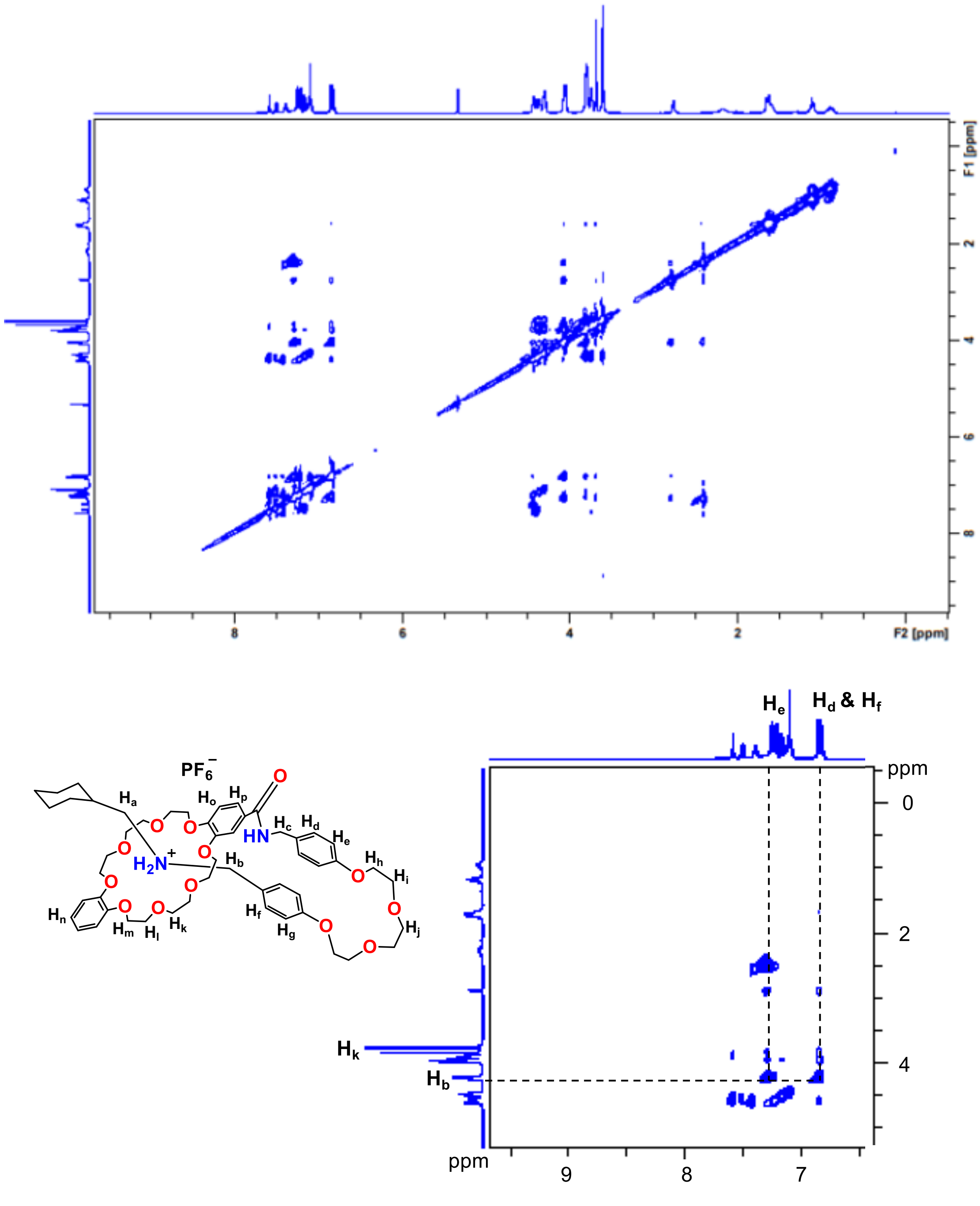

Figure S20. (Upper) Full NOESY spectrum (400 MHz, CD_2Cl_2, 298 K) of **1-H(Rot)·PF_6**. (Lower) Partial 2D NOESY spectrum (400 MHz, CD_2Cl_2, 298 K) of **1-H(Rot)·PF_6** shows the NCH_2 (H_b) protons correlate to aromatic protons H_d (H_b/H_d) and aromatic protons H_e (H_b/H_e).

4. Mass spectrum of 1-H(Rot)·PF_6

[1]Rotaxane **1-H(Rot)·PF_6** has also been characterized by Matrix-assisted laser desorption/ionization time-of-flight mass spectrometry (MALDI-TOF-MS) by a deprotonation of **1-H(Rot)·PF_6** with an excess amount of Et_3N. The deprotonated [1]rotaxane was relatively stable at room temperature.[3] A singly charged molecular ion base peak (*m/z*) of 953.4765 was observed which corresponded to the {[1]rotaxane+Na}$^+$ molecular ion with a theoretical value of 953.4770 (**Figure S20**). This result of MALDI-TOF-MS further confirmed the successful synthesis of [1]rotaxane without other species such as dimer, trimer, oligomer, etc.

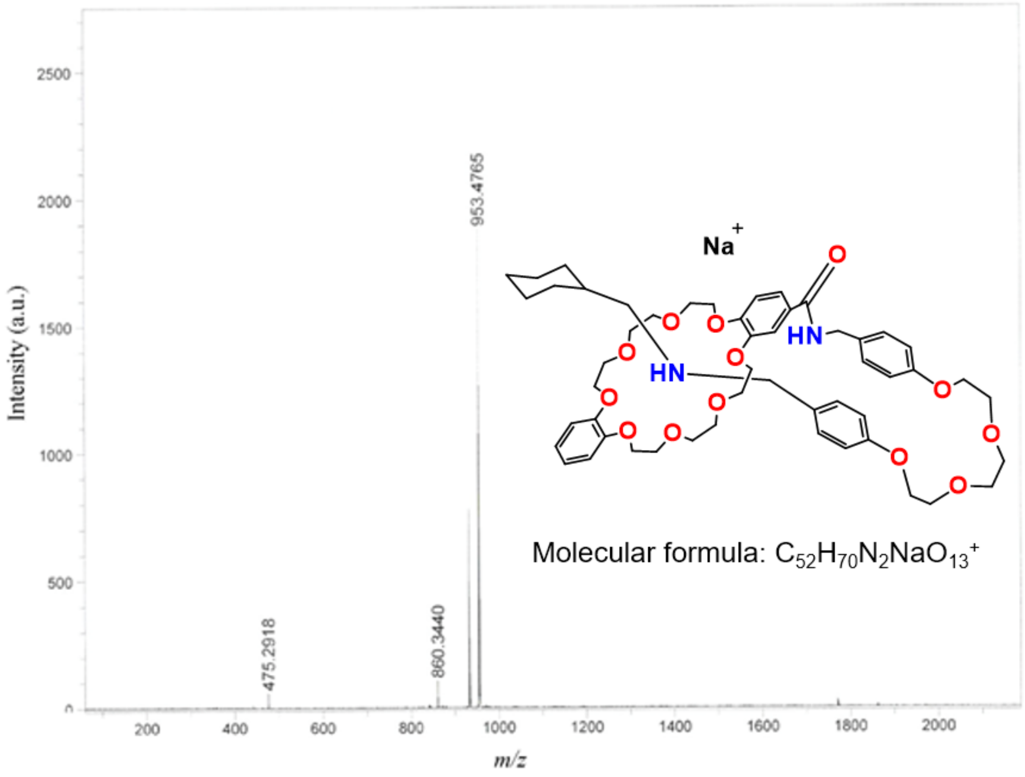

Figure S21. HRMS (MALDI-TOF) spectrum of **1-H(Rot)·PF_6**.

5. References

[1] Reproduced/adapted from: C.-H. Wong, "Synthesis, Characterization and Application of Thermo-responsive [1]Pseudorotaxane Prepared by Slippage Approach", PhD thesis, Hong Kong Baptist University, 2017.

[2] M. Han, H.-Y. Zhang, L.-X. Yang, Z.-J. Ding, R.-J. Zhuang, Y. Liu, *European Journal of Organic Chemistry* **2011**, *2011*, 7271-7277.

[3] K. C.-F. Leung, K.-N. Lau, W.-Y. Wong, *International Journal of Molecular Sciences* **2015**, *16*, 8254-8265.
